# The association between body mass index, abdominal fatness, and weight change and the risk of adult asthma: a systematic review and meta-analysis of cohort studies

**DOI:** 10.1038/s41598-023-31373-6

**Published:** 2023-05-12

**Authors:** Ganeshkumar Parasuaraman, Lavanya Ayyasamy, Dagfinn Aune, Abhijit Sen, Ramya Nagarajan, Prabhu Rajkumar, Saravanakumar Velusamy, P. Manickam, Satish Sivaprakasam

**Affiliations:** 1 Indian Council of Medical Research-National Institute of Epidemiology, Chennai, India; 2 Department of Nutrition, Oslo New University College, Oslo, Norway; 3 Department of Epidemiology and Biostatistics, School of Public Health, Imperial College London, London, UK; 4 Department of Endocrinology, Morbid Obesity and Preventive Medicine, Oslo University Hospital, Oslo, Norway; 5 Department of Public Health and Nursing, Norwegian University of Science and Technology, Trondheim, Norway; 6 Center for Oral Health Services and Research (TkMidt), Trondheim, Norway

**Keywords:** Medical research, Risk factors

## Abstract

Obesity has been associated with increased risk of adult asthma, however, not all studies have found a clear association between overweight and the incidence of asthma, and data on other adiposity measures have been limited. Hence, we aimed to summarize evidence on association between adiposity and adult asthma. Relevant studies were retrieved through searches conducted in PubMed, and EMBASE up to March 2021. A total of sixteen studies (63,952 cases and 1,161,169 participants) were included in the quantitative synthesis. The summary RR was 1.32 (95% CI 1.21–1.44, I^2^ = 94.6%, p_heterogeneity _< 0.0001, n = 13) per 5 kg/m^2^ increase in BMI, 1.26 (95% CI 1.09–1.46, I^2^ = 88.6%, p_heterogeneity_ < 0.0001, n = 5) per 10 cm increase in waist circumference and 1.33 (95% CI 1.22–1.44, I^2^ = 62.3%, p_heterogeneity_= 0.05, n = 4) per 10 kg increase in weight gain. Although the test for nonlinearity was significant for BMI (p_nonlinearity_ < 0.00001), weight change (p_nonlinearity_ = 0.002), and waist circumference (p_nonlinearity_ = 0.02), there was a clear dose-response relationship between higher levels of adiposity and asthma risk. The magnitude of the associations and the consistency of the results across studies and adiposity measures provide strong evidence that overweight and obesity, waist circumference and weight gain increases asthma risk. These findings support policies to curb the global epidemic of overweight and obesity.

## Introduction

Asthma is a major public health problem worldwide. According to the Global Burden of Disease Study, more than 339 million people had prevalent asthma, 420,000 deaths occurred and 24.8 million disability-adjusted life-years (DALYs) were lost due to asthma globally in 2016^1,2^. More than 80% of asthma-related deaths occur in low and lower-middle-income countries. The estimated asthma prevalence based on the World Health Survey (2002–2003) was 4.3% among the younger adults (18–45 years) who reported physician diagnosed asthma, and 4.5% reported either a physician’s diagnosis or that they were taking treatment for asthma, and 8.6% reported that they had experienced attacks of wheezing or whistling breath (symptoms of asthma) in the preceding 12 months^3^. Risk factors for asthma include genetic susceptibility, allergic sensitization, environmental tobacco smoke, air pollution, mould and damp, exposure to animals, use of medications (antibiotics and acetaminophen), and occupational exposures such as woodworking, farming, paint containing isocyanates, and diet (fast food)^2,4^.Thus, environmental factors are major drivers of the risk of developing asthma^2,4^.

Overweight (body mass index, BMI 25–< 30 kg/m^2^) or obesity (BMI ≥ 30) is defined as a weight that is higher than what is considered as a healthy weight for a given height. Globally, the age-standardized mean BMI increased from 21.7 to 24.2 kg/m^2^ between 1975 and 2015 among men, and it increased from 22.1 to 24.4 kg/m^2^ among women in the same period^5^. Currently, the global prevalence of obesity is 10.8% in men and 14.9% in women. It has been projected that if this trend continues, the prevalence of obesity will be 18% in men and 21% in women in 2025^5^. Although evidence from cohort studies suggests that there is a positive association between obesity and increased incidence of asthma^6–18^, studies have been less consistent with regards to whether overweight is associated with increased risk as some studies reported an increased risk^7–11,13–16,18^ and others found no association^6,17^. Most of the available studies have reported a dose–response relation between increasing BMI and asthma incidence, but studies have differed somewhat with regard to what BMI level risk of asthma starts to increase^6–21^. Fewer studies have been published on other measures of adiposity and risk of asthma, but four studies reported increased asthma risk with increasing waist circumference^7,11,13,21^, while three studies reported an increased risk with greater weight gain^10,16,22^, and a fourth study found no clear association^18^. In contrast, studies have generally reported no association between weight loss and asthma^10,16,18,22^. A meta-analysis published in 2007 including seven cohort studies found increased risk of asthma with overweight and obesity^23^, while a second meta-analysis found increased asthma risk with abdominal obesity^24^, however, dose–response analyses were not conducted, and no meta-analysis has been published on a range of adiposity measures and adult asthma risk. In addition, several additional cohort studies have since been published on BMI and adult asthma risk^6,7,11–16,21,22^. For these reasons, we conducted a systematic review and meta-analysis of cohort studies on the association between different measures of adiposity and the risk of developing asthma among adults, with particular aims of clarifying the strength and shape of the dose–response relationship, and to clarify potential confounding by conducting subgroup analyses by adjustment for smoking and other established risk factors.

## Methods

### Search strategy

This systematic review was registered in PROSPERO (CRD42020167990). We adopted a detailed search strategy with search terms as presented in Supplementary Table 1. With the search strategy, we systematically searched in PubMed and Embase databases for cohort studies that investigated the association between adiposity and asthma up to 16th March 2021. The search was carried out in mentioned databases by using both Medical Subject Headings (MeSH) terms related to our exposure and outcome and free-text words with appropriate truncations, wildcards, and proximity. Appropriate Boolean operators (“OR” and “AND”) were used to combine the individual search results. The search was limited to articles written in English. References of the included studies were searched for any potential further studies.

### Eligibility criteria

The eligibility criteria were framed using the PECO(S) elements^25^.

#### Population

Adults aged ≥ 18 years.

#### Exposure

Any measure of adiposity.

#### Comparator

People with the lowest level (or in the reference category) of adiposity across studies.

#### Outcome

Adult asthma.

### Study design

Prospective cohort studies, retrospective cohort studies, case-cohort studies, nested case–control studies within cohort studies, and retrospective cohort studies.

In addition, to the above criteria the studies had to report adjusted risk estimates (relative risks [RRs], odds ratios [ORs] or hazard ratios [HRs]) with 95% confidence intervals (CIs) for the association between adiposity and the risk of incident bronchial asthma. Retrospective case–control studies, cross-sectional studies, and case-only studies as well as studies in pregnant women were excluded. Only full-text articles were included, while conference abstracts, unpublished data, and grey literature were excluded.

### Study selection process

We adopted three stages in the study selection process.

#### Stage 1: Primary screening

All retrieved studies from data bases were imported to Mendeley and the list of selected studies to be screened was prepared by removing the duplicates. Two independent investigators (LA and RN) screened the titles, abstracts, and keywords of the identified citations. Full-text articles were retrieved from these shortlisted studies.

#### Phase 2: Secondary screening

Based on the eligibility criteria of our review, full-text articles were screened based on the selected articles from the primary screening by the same two reviewers (LA and RN). We excluded the studies which did not meet the eligibility criteria and exclusion reasons were noted.

#### Phase 3: Final study selection

During the screening process, conflicts on the selection of the studies between two investigators were resolved and the final studies were selected by consensus with two other reviewers (PG and DA). The list of excluded studies with exclusion reasons is provided in Supplementary Table 2.

### Data extraction

Data extraction from the selected studies was carried out independently by two reviewers (LA and RN) using a pre-designed data extraction form. When extractions differed between the reviewers, they were resolved by consensus. We extracted the name of the first author, publication year, geographic location, name of the study, follow-up period, sample size, age, sex, number of cases, the method for assessment of adiposity (self-report vs. measured), adiposity variable, quantity of the adiposity exposure, relative risk estimates and corresponding 95% CIs, and confounders adjusted for in the analysis from each study.

### Study quality assessment

We adopted the Newcastle–Ottawa (NO) scale for cohort studies for assessing the quality of all included studies^26^. It was done by two independent reviewers (LA and RN). Study quality was assessed on the following domains; selection, exposure, comparability, and outcome. We modified the Newcastle Ottawa scale for this review similarly as in a recent meta-analysis^27^ by excluding the point about representability as this is not directly a measure of study quality, and by modifying the score for the questions on adjustment for confounding factors, as the original scale gave 1 point for each of two confounding factors adjusted for, however, a study with adjustment only for age and sex would receive a maximum score, but could still be confounded by other risk factors. A score of 0.25 was given for each confounding factor that the analyses were adjusted for, up to a maximum of two points for eight or more confounders that were adjusted for. The score for record linkage was modified to 0.5 instead of 1, and a 1 point was given if the outcome assessment had been validated. This gave a total score of between 0 and 8 which was further grouped as 0–3 (low quality), > 3–6 (medium quality) and > 6–8 (high quality).

### Evidence grading

We used World Cancer Research Fund criteria for evaluating the likelihood of causality for the observed associations between adiposity and risk of adult asthma according to predefined criteria^28^. The system grades evidence ranging from substantial effect on risk unlikely, limited—no conclusion, limited-suggestive, probable, to convincing.

### Statistical analysis

We used a random effects model to estimate summary RRs and 95% CIs for the association between BMI, waist circumference, and weight change (the three exposures with a sufficient number of studies for statistical analyses) and asthma. We adopted the method of Greenland and Longnecker to estimate study-specific linear trends across categories of BMI, waist circumference, and weight change^29^. For studies reporting median or mean BMI, waist circumference, or weight change per category these values were used as reported, while for studies reporting a range of BMI, waist circumference, or weight change, the average of the lower and upper cut-off points was used as the midpoint. When extreme ranges were open-ended, we estimated an upper or lower cut-off value based on the width of the adjacent interval. When the reference category was not the lowest, we used Hamling's approach to recalculate the risk estimates so the lowest category became the reference category^30^. We used a BMI of 15 as a lower bound for underweight and 18.5 as a lower bound for normal weight where studies provided analyses by WHO categories of BMI. We used fractional polynomial models for examining the nonlinear dose–response relationships between BMI, waist circumference, weight change and asthma^31^. The model with the lowest deviance was found to be the best fit second-order fractional polynomial regression model. A likelihood ratio test was used to assess differences between the linear and nonlinear models in order to test for nonlinearity.

Heterogeneity was evaluated by chi-square and I^2^ statistics^32^. We considered a p-value < 0.10 in chi-square test as indication of significant heterogeneity, while I^2^ was used to quantify the proportion of total variability due to between-study heterogeneity, ranging from 0 to 100%. We tested for publication bias using Egger’s test^33^, Begg’s test^34^, and by visually inspecting the funnel plots when there was at least ten studies included in the analysis. A p-value < 0.10 with Egger’s test was considered as an indication of potential publication bias. Sensitivity analyses were conducted to test whether the overall association was explained by a study with an extreme result or a very large study, which would have a large influence on the overall summary estimate. In these sensitivity analyses, we reported the summary estimates when excluding the studies with the most positive and negative impact on the results. We calculated E-values for the association between adiposity variables and asthma, to assess the potential impact of unmeasured or uncontrolled confounding. The E-value is defined as the lowest strength that an uncontrolled or unmeasured confounder would have with both the exposure and the outcome to fully explain away the observed association^35^. The analyses were conducted using Stata 13.1 (StataCorp, College Station, TX, USA).

## Results

**Figure 1:**
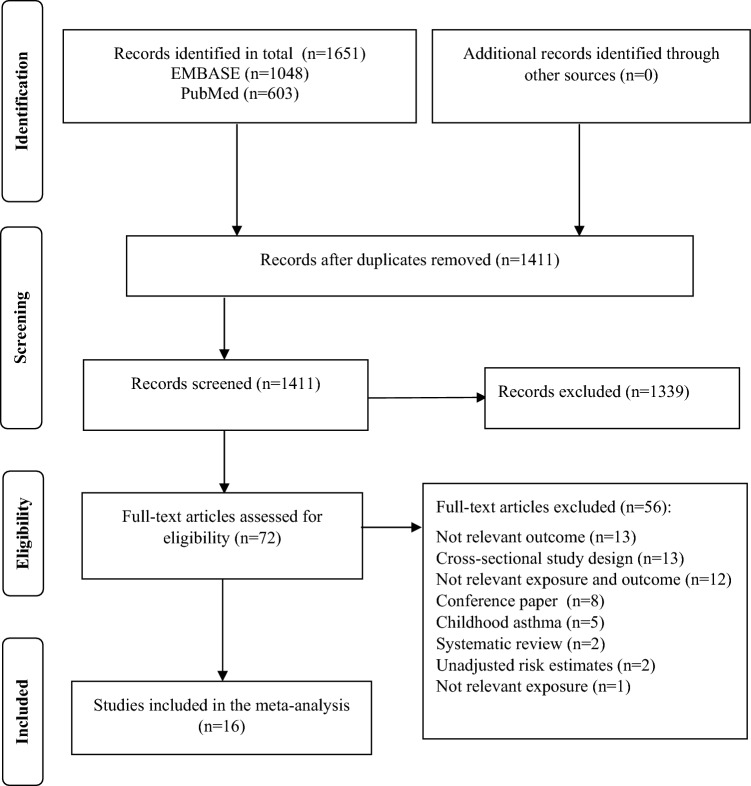
PRISMA flow chart.

We identified 1651 studies through the systematic literature search of PubMed (n = 1048) and EMBASE (n = 603) databases (Fig. 1). After removing the duplicates, 1411 studies were screened during the primary screening stage. We deemed 72 of those studies relevant for the full-text retrieval. During the second screening stage, we excluded 56 studies which did not meet the eligibility criteria. In total, 16 studies were included in our review^6–18,21,22,36^.

### Characteristics of the included studies

The characteristics of the sixteen included studies are presented in Table 1. Of these, seven studies were from North America (6 from the US and 1 from Canada), five were from Europe (two from Norway, two from France, and one from Finland), three studies were from Asia (one each from Korea, Japan and China), and one study was from Australia. In terms of gender inclusion, three studies were conducted among females and twelve studies were conducted among both males and females. The number of study participants ranged from 4532 to 246,361 and the duration of follow-up ranged from 2 to 25 years.

**Table 1:** Characteristics of included studies (N = 16).

Author name, publication year, country	Study name	Follow-up period	Study size	Sex	Age	Number of cases	Assessment of height and weight	Exposure and subgroup	Comparison	RR (95% CI)	Adjustment for confounders
Camargo CA et al., 1999, USA	The Nurses’ Health Study II	1991–1995, 4 years follow up	85911	Women	26–46 years	1596	Self-reported (validated)	BMI	<20 kg/m^2^	0.9 (0.7–1.1)	Age, race, US region, smoking status, physical activity, total energy intake, hysterectomy status, birth weight, and duration of breastfeeding
									20.0–22.4	1.0	
									22.5–24.9	1.1 (1.0–1.3)	
									25.0–27.4	1.6 (1.3–1.9)	
									27.5–29.9	1.7 (1.4–2.0)	
									≥30.0	2.7 (2.3–3.1)	
								Weight change since age 18 years	<− 5 kg	0.8 (0.6–1.1)	
									− 5 to − 2.1	0.8 (0.6–1.1)	
									− 2 to 2	1.0	
									2.1 to 5	0.9 (0.8–1.2)	
									5.1 to 10	1.1 (0.9–1.3)	
									10.1 to 20	1.4 (1.2–1.7)	
									20.1 to 25	2.0 (1.6–2.5)	
									>25	2.5 (2.0–3.1)	
Beckett WS et al., 2001, USA	CARDIA study	1986–1996, 10 years follow-up	4532	Men and women	18–30	434	Measured	BMI percentiles	1–19	1.09 (0.78–1.51)	Age, race, sex, centre, and maximal education
									20–39	1.00	
									40–59	0.99 (0.71–1.37)	
									60–79	1.10 (0.80–1.53)	
									80–100	1.21 (0.88–1.68)	
Chen Y et al, 2002, Canada	Canadian National Population Health Surveys	1994–1996, 2 years follow-up	4883	Women	20–64 years	127	Self-reported	BMI	<20.0 kg/m^2^	0.62 (0.24–1.62)	Age, smoking, pet(s) at home, immigrant status, history of allergy, income adequacy, and alcohol drinking
									20.0–24.9	1.00	
									25.0–29.9	1.25 (0.72–2.18	
									≥30.0	1.92 (1.09–3.41)	
Huovinen E et al., 2003, Finland	The Finnish Twin Cohort	1982–1990, 9 years follow-up	10,597	Men and women	25–52 years	149	Self-reported	BMI, men	<20.0 kg/m^2^	0.41 (0.06–3.06)	Age, atopy and respiratory symptoms
									20.0–<25	1.00	
									≤25–30	1.07 (0.62–1.85)	
									≥30.0	3.47 (1.56–7.72)	
								BMI, women	<20.0 kg/m^2^	1.56 (0.87–2.78)	
									20.0–<25	1.00	
									≤25–30	1.60 (0.91–2.80)	
									≥30.0	2.30 (0.87–6.08)	
								Weight change, men	<− 2 kg	0.72 (0.31–1.63)	
									<2	1.00	
									2 to 10	0.98 (0.56–1.74)	
									>10	1.41 (0.50–3.98)	
								Weight change, women	<− 2 kg	1.12 (0.57–2.21)	
									<2	1.00	
									2 to 10	0.95 (0.57–1.59)	
									>10	1.45 (0.54–3.90)	
Romieu I et al., 2003, France	E3N Cohort Study	1990–1993, 3 years follow-up	67229	Women	40–60 years	372	Self-reported (not validated)	BMI	<20.20 kg/m^2^	0.93 (0.66–1.31)	Age, total caloric intake, physical activity, smoking status, and menopausal status.
									20.20–21.41	1.00	
									21.42–22.68	1.22 (0.88–1.69)	
									22.69–24.61	1.18 (0.84–1.64)	
									24.62–26.99	1.46 (1.01–2.12)	
									≥27	2.02 (1.38–2.98)	
Ford ES et al., 2004, USA	First National Health and Nutrition Examination Survey	1971–1974–1982–1984, 10 years follow-up	14407	Men and women	25–74 years	317	Measured	BMI, all	18.5–<25.0 kg/m^2^	1.00	Age, sex (except sex-specific models), race or ethnicity, education, smoking status, recreational physical activity and nonrecreational activity
									25.0–<30.0	0.95 (0.68–1.33)	
									30.0–<35.0	1.28 (0.83–1.96)	
									≥35.0	1.87 (1.12–3.13)	
								BMI, men	18.5–<25.0 kg/m^2^	1.00	
									25.0–<30.0	0.79 (0.47–1.34)	
									30.0–<35.0	1.52 (0.82–2.82)	
									≥35.0	1.98 (0.60–6.54)	
								BMI, women	18.5–<25.0 kg/m^2^	1.00	
									25.0–<30.0	1.13 (0.73–1.75)	
									30.0–<35.0	1.10 (0.64–1.89)	
									≥35.0	1.87 (1.05–3.33)	
Coogan PF et al, 2009, USA	Black Women’s Health study	1995–2005, 10 years follow-up	46,435	Women	21–69 years	1068	Measured	BMI	<20 kg/m^2^	0.84 (0.56–1.26)	Adjusted for age, time period; current smoker, pack-years of smoking, second hand smoke, parental history of asthma, menopausal status, marital status, income, menopausal female hormone use, metabolic equivalents, BMI at age 18 years, energy intake
									20–24	1.00	
									25–29	1.26 (1.05–1.51)	
									30–34	1.62 (1.32–1.98)	
									35–39	2.24 (1.76–2.84)	
									≥40	2.85 (2.19–3.72)	
								Weight change since age 18 years	<0 kg	1.01 (0.72–1.41)	
									0–9	1.00	
									10–14	1.07 (0.84–1.37)	
									15–19	1.29 (1.02–1.63)	
									20–24	1.35 (1.07–1.71)	
									≥25	1.88 (1.54–2.28)	
Hjellvik V et al., 2010, Norway	Norwegian Health Survey	2004–2007, 3 years follow up	118723	Men and women	40 years	4049	Measured	BMI	<20 kg/m^2^	1.0 (0.9–1.2)	Age, sex, education, physical activity, disability pension, urban/rural status, smoking status
									20–24.9	1.0	
									25–29.9	1.3 (1.2–1.4)	
									30–34.9	1.8 (1.6–2.0)	
									≥35	2.4 (2.0–2.9)	
Korda RJ et al., 2012, Australia	45 and Up Study	2006–2009, 2.3 years follow up	246361	Men and women	45-≥80 years	275	Self-reported data (validated)	BMI, age 45–64 years	18.5–<25 kg/m^2^	1.00	Age, sex, area of residence, education, pre-tax annual household income, smoking, alcohol intake, private health insurance
									25.0–<30	2.30 (1.41–3.75)	
									≥30	3.97 (2.48–6.36)	
								BMI, age 65–79 years	18.5–<25 kg/m^2^	1.00	
									25.0–<30	0.84 (0.50–1.41)	
									≥30	1.64 (0.98–2.73)	
								BMI, age≥80 years	18.5–<25 kg/m^2^	1.00	
									25.0–<30	1.69 (0.80–3.56)	
									≥30	3.06 (1.35–6.94)	
Brumpton B et al., 2012, Norway	HUNT study	1997–2008, 11 years follow-up	23245	Men and women	19–55 years	818	Measured	BMI, women	25.0 kg/m^2^	1.00	Age, smoking, physical activity, education, family history
									25.0–29.9	1.44 (1.18–1.76)	
									≥30.0	1.96 (1.52–2.52)	
									Per 5 kg/m^2^	1.32 (1.19–1.45)	
								BMI, men	25.0 kg/m^2^	1.00	
									25.0–29.9	1.21 (0.93–1.58)	
									≥30.0	1.84 (1.30–2.59)	
									Per 5 kg/m^2^	1.38 (1.17–1.62)	
								WC, Women	<80.0 cm	1.00	
									80–87.9	1.30 (1.04–1.62)	
									≥88	1.88 (1.51–2.34)	
									Per 10 cm	1.28 (1.18–1.38)	
								WC, men	<80.0 cm	1.00	
									80–87.9	1.15 (0.87–1.52)	
									≥88	1.55 (1.08–2.21)	
									Per 10 cm	1.23 (1.08–1.41)	
Leone N et al., 2012, France	The 3C study	NA-NA, 4 years follow-up	6267	Men and women	>65 years	67	Measured	WC	<94/80 cm	1.00	Age, centre, educational level, smoking status, physical ability, cardiovascular disease history, dyspnoea, and chronic bronchitis symptoms.
									94/80–102/88	2.69 (1.21–5.98)	
									≥102/88	3.84 (1.55–9.49)	
Assad N et al, 2013, USA	CARDIA study	1985–2010, 25 years follow-up	4619	Men and women	Mean age 24.9 years	602	Measured	BMI, all	Per 5 kg/m^2^	1.14 (1.06–1.22)	Age, race, smoking, and physical activity
								BMI, men	Per 5 kg/m^2^	1.08 (0.92–1.27)	
								BMI, women	Per 5 kg/m^2^	1.15 (1.06–1.26)	
Tomita Y et al, 2018, Japan	Health insurance claims and health checkups	2012–2015, ~3 years follow-up	9888	Men and women	40–64 years	424	Measured	BMI, men	<18.4 kg/m^2^	0.64 (0.20 - 2.04)	Age, smoking status, allergic rhinitis, and metabolic syndrome
									18.5–24.9	1.00	
									25–29.9	1.10 (0.76 - 1.59)	
									≥30.0	0.94 (0.44 - 1.98)	
								BMI, women	<18.4 kg/m^2^	1.03 (0.66 - 1.59)	
									18.5–24.9	1.00	
									25–29.9	2.02 (1.38 - 2.96)	
									≥ 30.0	2.65 (1.26 - 5.54)	
								WC, men	≤ 84.9 cm	1.00	
									85–89.9	0.97 (0.59 - 1.60)	
									≥ 90	0.89 (0.52 - 1.53)	
								WC, women	≤ 84.9 cm	1.00	
									85–89.9	1.34 (0.87 - 2.07)	
									≥ 90	2.33 (1.37 - 3.96)	
Park S et al., 2019, Korea	National Health Insurance Service-Health Screening Cohort	2004–2013, 9 years follow-up	459529	Men and women	≥40 years	53371	Measured	BMI, men	<18.5 kg/m^2^	1.32 (1.23–1.43)	Age, insurance type, household income, smoking, drinking, physical activity
									18.5–< 23	1.00	
									23–< 25	1.00 (0.96–1.03)	
									25–< 30	1.04 (1.07–1.07)	
									≥ 30.0	1.23 (1.13–1.34)	
								BMI, women	<18.5 kg/m^2^	0.94 (0.87–1.03)	
									18.5–<23	1.00	
									23–<25	1.11 (1.07–1.14)	
									25–<30	1.24 (1.21–1.28)	
									≥30.0	1.40 (1.32–1.48)	
								WC, men	<80 cm	1.00	
									80–<90	1.05 (0.99–1.12)	
									90–<100	1.13 (1.05–1.21)	
									≥100	1.34 (1.16–1.57)	
								WC, women	<75 cm	1.00	
									75–<85	1.14 (1.08–1.21)	
									85–<95	1.23 (1.14–1.32)	
									≥95	1.19 (1.03–1.37)	
Wang H et al, 2020, China	Shandong Multi-Center Health Check-up Longitudinal Study	1999–2000–2015–2016, 12 years follow-up	42304	Men and women	≥20 years	90	Measured	BMI, men	<25 kg/m^2^	1.00	Age, smoking and drinking status
									25–<30	0.78 (0.44–1.39)	
									≥30	0.77 (0.27–2.21)	
								BMI, women	<25 kg/m^2^	1.00	
									25–<30	2.71 (1.31–5.61)	
									≥30	6.41 (2.34–17.54)	
Wang T et al., 2021, USA	National Health and Nutrition Examination Survey	1999–2000–2015–2016, 10 years follow-up	20771	Men and women	40–74 years	627	Measured height, self-reported recalled weight	Weight change since age 25 years	<− 2.5 kg	0.91 (0.53–1.56)	Age, sex, race/ethnicity, education, family income-poverty level, smoking status, family history of asthma
									− 2.5 to <2.5	1.00	
									2.5 to <10	0.99 (0.73–1.34)	
									10 to <20	1.19 (0.88–1.60)	
									≥20	1.53 (1.15–2.03)	

WC = waist circumference.

### Assessment of study quality

The quality of included study was assessed using the modified Newcastle Ottawa scale and is presented in Supplementary Table 3. Overall, the mean (median) study quality was 5.9 (6.0).

### BMI and adult asthma

**Figure 2:**
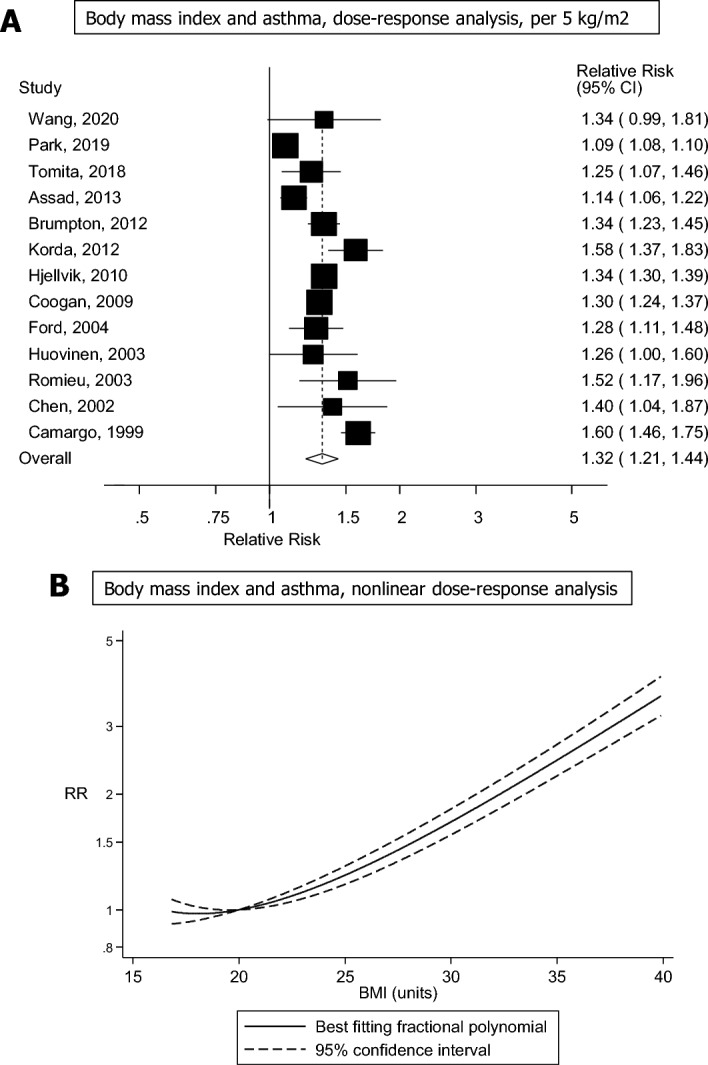
Forest plot showing the (**A**) linear and (**B**) nonlinear dose–response analysis between body mass index and adult asthma. The squares represent the relative risks (RRs) and the lines through the squares represent the confidence intervals, and the diamond at the bottom represents the summary RR (**A**).

A total of thirteen studies including 63,258 cases and 1,134,131 participants were included in the linear dose–response analysis of BMI and risk of adult asthma^6–18^, and thirteen studies were included in the high vs. low analysis^6–11,13–18,36^. The summary RR was 2.13 (95% CI 1.66–2.73, I^2^ = 87.9%, p_heterogeneity_ < 0.0001) for high vs. low BMI (Supplementary Fig. 1) and 1.32 (95% CI 1.21–1.44, I^2^ = 95.3%, p_heterogeneity_ < 0.0001) per 5 kg/m^2^ increase (Fig. 2A). In sensitivity analyses, excluding one study at a time, the summary RR ranged from 1.29 (95% CI 1.19–1.41) when removing the Nurses’ Health Study II^10^ to 1.34 (95% CI 1.27–1.43) when removing the CARDIA study^12^ (Supplementary Fig. 2). There was indication of publication bias with Egger’s test (p = 0.01) and by investigation of the funnel plot (Supplementary Fig. 3), but not with Begg’s test (p = 0.36), However, there was no indication of bias when excluding a large Korean study^7^, which found a much weaker association than the remaining studies, the summary RR was not substantially altered (Supplementary Fig. 2), and Egger’s test (p = 0.63) was no longer significant and there was also no longer asymmetry in the funnel plot (Supplementary Fig. 4). There was a nonlinear association between BMI and risk of adult asthma (p_nonlinearity_ < 0.0001), and the nonlinear curve was flat between BMI value of 19 and 21 and increased at above this range (Fig. 2B, Supplementary Table 4), and the corresponding E-values were very strong (Supplementary Table 4).

#### Waist circumference and adult asthma

**Figure 3:**
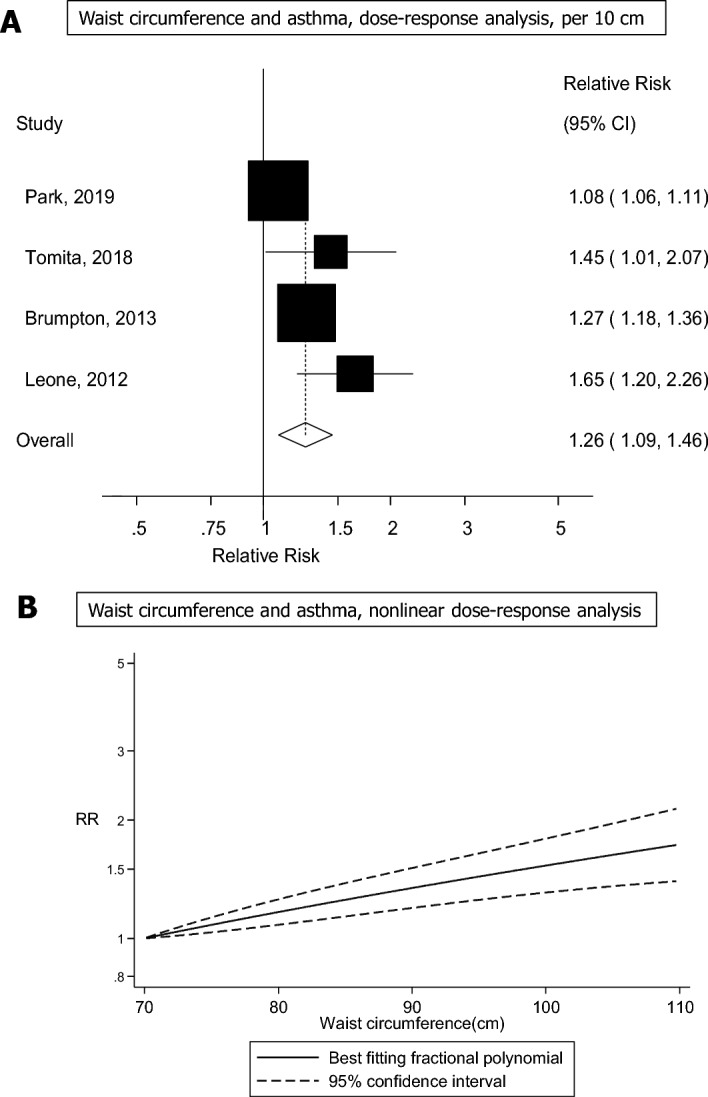
Forest plot showing the linear and nonlinear dose–response analysis between waist circumference and adult asthma. The squares represent the relative risks (RRs) and the lines through the squares represent the confidence intervals, and the diamond at the bottom represents the summary RR (**A**).

Four studies including 14,081 cases and 282,138 participants were included in the quantitative synthesis of waist circumference and asthma^7,11,13,21^. The summary RR was 1.56 (95% CI 1.22–2.01, I^2^ = 77.8%, p_heterogeneity_ = 0.004) for high vs. low waist circumference (Supplementary Fig. 5) and 1.26 (95% CI 1.09–1.46, I^2^ = 88.6%, p_heterogeneity_ =  < 0.0001) per 10 cm increase in waist circumference (Fig. 3A). The summary RR ranged from 1.20 (95% CI 1.04–1.39) when removing the study by Leone et al.^21^ to 1.35 (95% CI 1.16–1.57) when removing the study by Park et al.^7^ (Supplementary Fig. 6). Although the test of nonlinearity between waist circumference and asthma risk was significant (p_nonlinearity_ = 0.02), the association was dose-dependent and nearly linear (Fig. 3B, Supplementary Table 5), and the corresponding E-values were very strong (Supplementary Table 5).

#### Weight change and adult asthma

**Figure 4:**
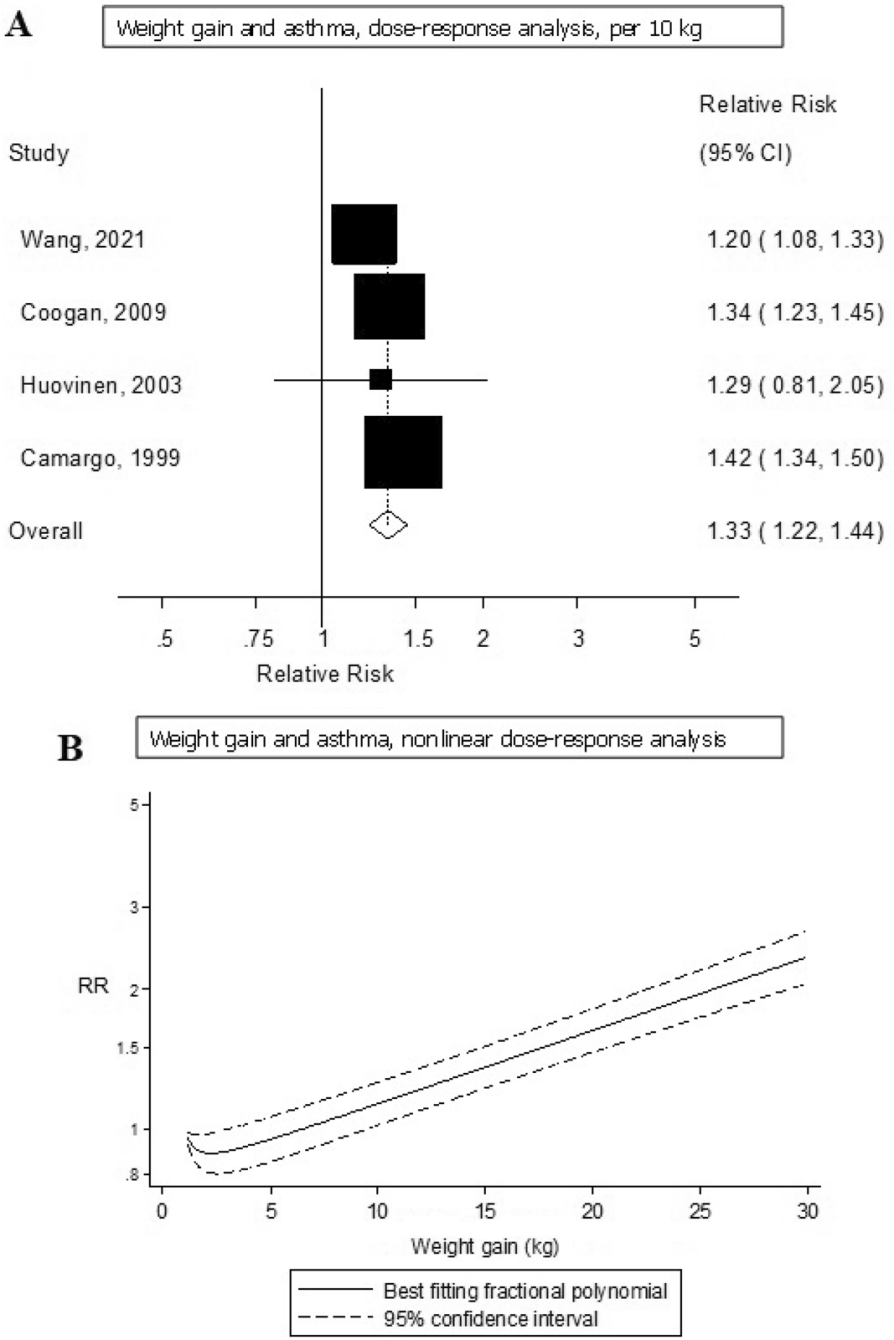
Forest plot showing the linear and nonlinear dose–response analysis between weight gain and adult asthma. The squares represent the relative risks (RRs) and the lines through the squares represent the confidence intervals, and the diamond at the bottom represents the summary RR (**A**).

**Figure 5:**
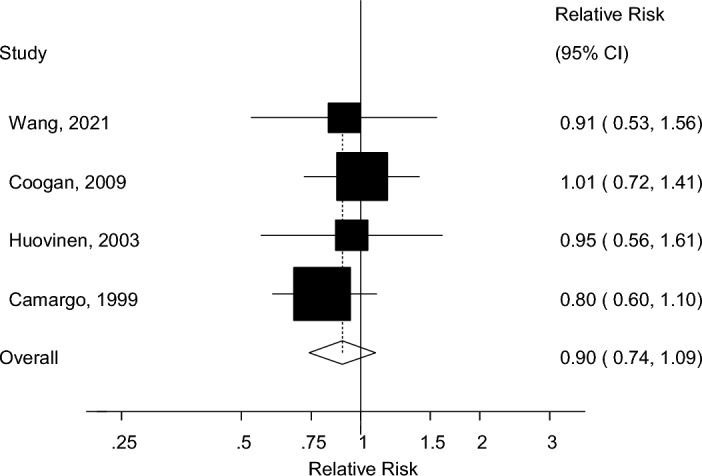
A forest plot depicting the relationship between weight loss and asthma risk. The squares represent the relative risks (RRs) and the lines through the squares represent the confidence intervals, and the diamond at the bottom represents the summary RR (Fig. 5).

Four studies including 3440 cases and 163,714 participants were included in the quantitative synthesis of weight gain and weight loss and asthma risk^10,16,18,22^. The summary RR was 1.93 (95% CI 1.53–2.44, I^2^ = 62.3%, p_heterogeneity_ = 0.05) for high vs. low weight gain (Supplementary Fig. 7) and 1.33 (95% CI 1.22–1.44, I^2^ = 62.3%, p_heterogeneity_ = 0.047) per 10 kg increase in weight gain (Fig. 4A). The summary RR ranged from 1.28 (95% CI 1.18–1.38) when excluding the study by Camargo et al.^10^ to 1.39 (95% CI 1.33–1.46) when excluding the study by Wang et al.^22^ (Supplementary Fig. 8). There was evidence of a nonlinear association between weight gain and asthma risk (p_nonlinearity_ = 0.002), with a slightly J-shaped curve, however, the association was approximately linear from a weight gain of around 2 kg and above (Fig. 4B, Supplementary Table 6), and the corresponding E-values were very strong (Supplementary Table 6). We could not perform dose–response analyses of weight loss and asthma risk, but a high vs. low comparison gave a summary RR of 0.90 (95% CI 0.74–1.09, I^2^ = 0%, p_heterogeneity_ = 0.78) for weight loss in relation to asthma risk based on data from 4 cohort studies^10,16,18,22^ (Fig. 5) and in sensitivity analyses that removed one study at a time, there was minimal variation in the summary estimate (Supplementary Fig. 9).

#### Subgroup and sensitivity analyses

We conducted subgroup analyses based on duration of follow-up, sex, geographic location, method for assessment of weight and height, number of cases, study quality as well as adjustment for a range of confounding factors. The positive association between BMI and asthma was observed across nearly all the subgroup and there was little evidence of heterogeneity between subgroups with meta-regression analyses (Supplementary Table 7). The only subgroup analyses with significant heterogeneity between subgroups were the subgroup analyses stratified by method of assessment of weight and height, where there was a weaker association among studies using measured vs. self-reported measures of weight and height and a stronger association among studies with a high study quality score vs. studies with a medium study quality score. The association between BMI and asthma was statistically significant in women, but not in men, however, there was no significant heterogeneity between subgroups and the association in men was borderline significant (Supplementary Table 7). Although heterogeneity within subgroups was in general high to very high, the observed heterogeneity was lower among the subgroups of European studies, studies with self-reported weight and height, lower number of cases, and with adjustment for allergy or education (Supplementary Table 7).

#### Evidence grading

Using the World Cancer Research Fund/American Institute for Cancer Research (WCRF/AICR) criteria for evaluating strength of evidence (likelihood of causality) (Supplementary Table 8), we considered the evidence for the association between higher BMI and increased risk of asthma to be convincing, and there was limited-suggestive evidence that greater waist circumference and weight gain increases the risk of asthma (Supplementary Tables 9, 10). There was limited evidence and no conclusion could be drawn with regard to the impact of weight loss on asthma risk (Supplementary Tables 9, 10).

## Discussion

In this dose–response meta-analysis of multiple measures of adiposity and the risk of asthma, we found a 32% increase in the RR of asthma per 5 units increase in BMI, a 26% increase in RR per 10 cm increase in waist circumference, and a 33% increase in RR per 10 kg increase in weight gain. Weight loss was not significantly associated with reduced risk, but statistical power may have been limited to detect a weak association. There was a strong positive dose–response relationship between increasing BMI and risk of asthma, with RRs of 1.23, 1.69, 2.45 and 3.64 at BMI values of 25, 30, 35, and 40 compared with the reference BMI of 20, and there was some indication of increased risk even within the high range of the normal weight category. The association between higher BMI and asthma persisted across most subgroup analyses and there was little indication of significant between subgroup heterogeneity with meta-regression analyses, except for a stronger association in studies with higher study quality. The association was statistically significant in women, but not in men, however, the association in men was borderline significant and there was no significant between subgroup heterogeneity. There was also a strong positive dose–response relationship between greater waist circumference and weight gain and the risk of asthma.

Our findings are consistent with two earlier meta-analyses on BMI^23^ and abdominal obesity^24^ and asthma risk. However, the present meta-analysis included a larger number of studies on BMI (13 vs. 7 studies), and was restricted to cohort studies to avoid or reduce the impact of recall bias, selection bias and reverse causation, while a previous meta-analysis also included case–control and cross-sectional studies^24^. In addition, we also conducted linear and nonlinear dose–response analyses to clarify the dose–response relationship between BMI, waist circumference and weight gain and risk of asthma. Other studies have reported that higher BMI is associated with narrowing of the airways^37^, reduced lung volume^38^ and lung function^39^ and one study found that greater duration of obesity was associated with reduced lung function^40^. Therefore, obesity is considered an important determinant of impaired lung function^40^. Further support for this is found in a trial conducted among obese patients with severe asthma and in a review which found a benefit of weight loss interventions in relation to asthma control^41,42^. Although we did not detect a significant inverse association for weight loss in relation to asthma risk in the current analysis, the summary estimate was in the direction of reduced risk and it is possible that the weight loss observed in the studies was too small to detect an association and/or that an association could have been more clearly demonstrated if the analyses of weight loss had been conducted among subjects who were overweight or obese initially. Another issue that potentially could have affected the results is whether the observed weight loss was intentional or not.

In adults, obesity produces considerable alterations in normal lung physiology, including the accumulation of fat in the thoracic and abdominal cavities, resulting in lung compression and a reduction in lung volume. Increased adipose tissue inflammation causes airway hyperresponsiveness (a distinguishing feature of asthma) that leads to increased risk of asthma in adults^43^. Obesity is associated with a chronic inflammatory response. It has been shown that adipose tissue in obese individuals infiltrates with macrophages that promote and secrete systemic pro-inflammatory mediators such as tumor necrosis factor-α, leptin, but reduces adiponectin, and this causes inflammation in the airway, and leads to increases airway hyperresponsiveness and may contribute to asthma in persons who are obese^44^. Obesity also alters lung mechanics which contribute to symptoms of dyspnoea that occurs in absence of the airway inflammatory changes which lead to an increased risk of asthma^45^. The hormone leptin partly plays a role as indirect mediator of the association between high body adiposity and persistent asthma over time^46^. Leptin is also involved in pathophysiological process of asthma through improved phagocytosis, activation, proliferation, and alteration of macrophages, and induced production of proinflammatory cytokines^47^. In obesity, adipokines and adiponectin levels are reduced, which lowers the pulmonary function, and causes airway hyperresponsiveness, and airway inflammation^48^. Visceral fat has more inflammatory cells than subcutaneous fat and visceral fat is the main source of proinflammatory cytokines that may promote airway inflammation or may influence the structures of airways such as smooth muscle cells, blood vessels, and nerves which leads to airway hyperresponsiveness (AHR) in asthma^49,50^.

The rigorous literature search was one of the strengths of our review and methodology. In addition, the detailed subgroup and sensitivity analyses, which showed that the results were consistent across major subgroups, robust to the influence of single studies as well as potential confounders, are additional strengths. The results were also consistent across geographic regions with different patterns of confounding factors. Moreover, to our knowledge this is the first meta-analysis to clarify the dose–response relationship between different measures of adiposity and asthma risk through both linear and nonlinear dose–response analyses. The current meta-analysis was based on data from cohort studies, and excluded data from cross-sectional and case–control studies. Therefore the results are less likely to have been affected by recall bias, selection bias and reverse causation. The studies included in our review were of medium to high quality.

This review also has some limitations. We can't rule out the possibility that confounding from other risk factors may have contributed to the observed associations, as in any observational study. However, this seems less likely as the observed association between BMI and asthma was consistent across most subgroups. In addition, the associations with the different adiposity variables were relatively strong, thus we consider it less likely that confounding could fully explain the observed associations. Based on the estimated RRs and the E-values we found that an unadjusted confounder would have to be very strongly associated with both the exposure (adiposity) and the outcome (asthma) to fully explain away the observed associations. For example RRs for an unadjusted confounder would have to be around 2 for overweight, and 3–9 for higher levels of BMI, waist circumference, and weight gain, to explain away the observed associations fully. Although there was heterogeneity between studies, this appeared to be due to differences in the strength of the association rather than differences in the direction of the association, since all the studies reported risk estimates in the direction of higher risk. This is much less of an issue than if there is heterogeneity in the direction of the risk estimates. In addition, there was lower heterogeneity in some subgroups including European studies, studies with self-reported weight and height (both validated and not validated), studies with a lower number of participants and cases and among studies with adjustment for allergy and education. Publication bias can be an issue in meta analyses of published studies. Although we did find some indication of publication bias in the primary analysis, the asymmetry in the funnel plot was driven by a large Korean study which showed a much weaker association compared to the remaining studies, and when excluded from the analysis, the test for publication bias and the asymmetry in the funnel plot did not persist, while the summary estimate was only slightly modified.

This meta-analysis found evidence that different measures of adiposity including higher BMI, waist circumference, and weight gain, were strongly associated with increased risk of asthma in adults. These findings provide strong evidence that adiposity is associated with increased risk of asthma and reinforce the importance of public health policies and interventions (e.g. to target dietary patterns and physical activity) to reduce the burden of excess weight in the general population for the prevention of asthma.

## Supplementary Information

Supplementary Information.

## Data Availability

The data generated and analysed during the current study are available within the manuscript (Table 1).

## References

[1] GBD 2016 Disease and Injury Incidence and Prevalence Collaborators. Global, regional, and national incidence, prevalence, and years lived with disability for 328 diseases and injuries for 195 countries, 1990–2016: a systematic analysis for the Global Burden of Disease Study 2016. *Lancet.* **390**(10100), 1211–1259 (2017). 10.1016/S0140-6736(17)32154-2 PMC5605509 28919117

[2] Global Asthma Network. *The Global Asthma Report Asthma Affects*. (2018).

[3] Moores G, Boulet LP, Gershon AS (2012). Global asthma prevalence in adults: Findings from the cross-sectional world health survey. BMC Public Health.

[4] Sio YY, Chew FT (2021). Risk factors of asthma in the Asian population: A systematic review and meta-analysis. J. Physiol. Anthropol..

[5] Di Cesare M, Bentham J, Stevens GA (2016). Trends in adult body-mass index in 200 countries from 1975 to 2014: A pooled analysis of 1698 population-based measurement studies with 19.2 million participants. Lancet.

[6] Wang H, Bai C, Yi M (2020). Metabolic syndrome and incident asthma in chinese adults: An open cohort study. Diabetes Metab. Syndr. Obes. Targets Ther..

[7] Park S, Jung SY, Kwon JW (2019). Sex differences in the association between asthma incidence and modifiable risk factors in Korean middle-aged and older adults: NHIS-HEALS 10-year cohort. BMC Pulm. Med..

[8] Romieu I, Avenel V, Leynaert B, Kauffmann F, Clavel-Chapelon F (2003). Body mass index, change in body silhouette, and risk of asthma in the E3N cohort study. Am. J. Epidemiol..

[9] Chen Y, Dales R, Tang M, Krewski D (2002). Obesity may increase the incidence of asthma in women but not in men: Longitudinal observations from the Canadian National Population Health Surveys. Am. J. Epidemiol..

[10] Camargo CA, Weiss ST, Zhang S, Willett WC, Speizer FE (1999). Prospective study of body mass index, weight change, and risk of adult- onset asthma in women. Arch. Intern. Med..

[11] Tomita Y, Fukutomi Y, Irie M (2019). Obesity, but not metabolic syndrome, as a risk factor for late-onset asthma in Japanese women. Allergol. Int..

[12] Assad N, Qualls C, Smith LJ (2013). Body mass index is a stronger predictor than the metabolic syndrome for future asthma in women. The longitudinal CARDIA study. Am. J. Respir. Crit. Care Med..

[13] Brumpton B, Langhammer A, Romundstad P, Chen Y, Mai XM (2013). General and abdominal obesity and incident asthma in adults: the HUNT study. Eur. Respir. J..

[14] Korda RJ, Liu B, Clements MS (2013). Prospective cohort study of body mass index and the risk of hospitalisation: Findings from 246,361 participants in the 45 and Up Study. Int. J. Obes..

[15] Hjellvik V, Tverdal A, Furu K (2010). Body mass index as predictor for asthma: A cohort study of 118,723 males and females. Eur. Respir. J..

[16] Coogan PF, Palmer JR, O’Connor GT, Rosenberg L (2009). Body mass index and asthma incidence in the Black Women’s Health Study. J. Allergy Clin. Immunol..

[17] Ford ES, Mannino DM, Redd SC, Mokdad AH, Mott JA (2004). Body mass index and asthma incidence among USA adults. Eur. Respir. J..

[18] Huovinen E, Kaprio J, Koskenvuo M (2003). Factors associated to lifestyle and risk of adult onset asthma. Respir. Med..

[19] Guerra S, Sherrill DL, Bobadilla A, Martinez FD, Barbee RA (2002). The relation of body mass index to asthma, chronic bronchitis, and emphysema. Chest.

[20] Kronander UN, Falkenberg M, Olle Z (2004). Prevalence and incidence of asthma related to waist circumference and BMI in a Swedish community sample. Respir. Med..

[21] Leone N, Courbon D, Berr C (2012). Abdominal obesity and late-onset asthma: Cross-sectional and longitudinal results: The 3C study. Obesity.

[22] Wang T, Zhou Y, Kong N, Zhang J, Cheng G, Zheng Y (2021). Weight gain from early to middle adulthood increases the risk of incident asthma later in life in the United States: A retrospective cohort study. Respir. Res..

[23] Beuther DA, Sutherland ER (2007). Overweight, obesity, and incident asthma: A meta-analysis of prospective epidemiologic studies. Am. J. Respir. Crit. Care Med..

[24] Jiang D, Wang L, Bai C, Chen O (2019). Association between abdominal obesity and asthma: A meta-analysis. Allergy Asthma Clin. Immunol..

[25] Morgan RL, Whaley P, Thayer KA, Schünemann HJ (2018). Identifying the PECO: A framework for formulating good questions to explore the association of environmental and other exposures with health outcomes. Environ. Int..

[26] Wells, G., Shea, B., Robertson, J., Peterson, J., Welch, V., Losos, M. The Newcastle–Ottawa Scale (NOS) for assessing the quality of nonrandomized studies in meta-analysis bias and confounding Newcastle–Ottowa Scale. *Ottawa Hosp. Res. Inst*. 2012. http://www.evidencebasedpublichealth.de/download/Newcastle_Ottowa_Scale_Pope_Bruce.pdf.

[27] Halvorsen RE, Elvestad M, Molin M, Aune D (2021). Fruit and vegetable consumption and the risk of type 2 diabetes: A systematic review and dose-response meta-analysis of prospective studies. BMJ Nutr. Prev. Health..

[28] World Cancer Research Fund International. *Diet, Nutrition, Physical Activity and Cancer: A Global Perspective*. 2018. http://dietandcancerreport.org.

[29] Longnecker MP (1992). Methods for trend estimation from summarized dose-response data, with applications to meta-analysis. Am. J. Epidemiol..

[30] Hamling J, Lee P, Weitkunat R, Amb M (2007). Facilitating meta-analyses by deriving relative effect and precision estimates for alternative comparisons from a set of estimates presented by exposure level or disease category. Stat. Med..

[31] Bagnardi V, Zambon A, Quatto P, Corrao G (2004). Flexible meta-regression functions for modeling aggregate dose-response data, with an application to alcohol and mortality. Am. J. Epidemiol..

[32] Higgins JPT, Thompson SG (2002). Quantifying heterogeneity in a meta-analysis. Stat. Med..

[33] Egger M, Smith GD, Schneider M, Minder C (1997). Bias in meta-analysis detected by a simple, graphical test. BMJ.

[34] Begg CB, Mazumdar M (1994). Operating characteristics of a rank correlation test for publication bias. Biometrics.

[35] Van Der Weele TJ, Ding P (2017). Sensitivity analysis in observational research: Introducing the E-Value. Ann. Intern. Med..

[36] Beckett, W. S., Jacobs Jr., D. R., Yu, X., Iribarren, C. & Williams, O. D. Asthma is associated with weight gain in females but not males, independent of physical activity. *Am J Respir Crit Care Med.* **164**(11), 2045–2050 (2001). 10.1164/ajrccm.164.11.2004235 11739133

[37] Martin SE, Mathur R, Marshall I, Douglas NJ (1997). The effect of age, sex, obesity and posture on upper airway size. Eur. Respir. J..

[38] King GG, Brown NJ, Diba C (2005). The effects of body weight on airway calibre. Eur. Respir. J..

[39] Spathopoulos D, Paraskakis E, Trypsianis G (2009). The effect of obesity on pulmonary lung function of school aged children in Greece. Pediatr. Pulmonol..

[40] Santamaria F, Montella S, Greco L (2011). Obesity duration is associated to pulmonary function impairment in obese subjects. Obesity (Silver Spring).

[41] Dias-Júnior SA, Reis M, De Carvalho-Pinto RM, Stelmach R, Halpern A, Cukier A (2014). Effects of weight loss on asthma control in obese patients with severe asthma. Eur. Respir. J..

[42] Eneli IU, Skybo T, Camargo CA (2008). Weight loss and asthma: A systematic review. Thorax.

[43] Peters U, Dixon AE, Forno E (2019). Obesity and asthma. J Allergy Clin Immunol..

[44] Shore SA (2010). Obesity, airway hyperresponsiveness, and inflammation. J. Appl. Physiol..

[45] Sutherland ER (2014). Linking obesity and asthma. Ann. N. Y. Acad. Sci..

[46] Li Z, Leynaert B, Dumas O (2019). Role of leptin in the association between body adiposity and persistent asthma: A longitudinal study. Obesity.

[47] Tilg H, Moschen AR (2006). Adipocytokines: Mediators linking adipose tissue, inflammation and immunity. Nat. Rev. Immunol..

[48] Shore SA (2008). Obesity and asthma: Possible mechanisms. J. Allergy Clin. Immunol..

[49] Bruun JM, Lihn AS, Pedersen SB, Richelsen B (2005). Monocyte chemoattractant protein-1 release is higher in visceral than subcutaneous human adipose tissue (AT): Implication of macrophages resident in the AT. J. Clin. Endocrinol. Metab..

[50] Lommatzsch M (2012). Airway hyperresponsiveness: New insights into the pathogenesis. Semin. Respir. Crit. Care Med..

