# Supplementary material for: The association between body mass index, abdominal fatness, and weight change and the risk of adult asthma: a systematic review and meta-analysis of cohort studies
Source: Sci Rep. 2023 May 12;13:7745. doi: 10.1038/s41598-023-31373-6 (PMC10182066; doi:10.1038/s41598-023-31373-6)
Supplement: Supplementary file 1 — Supplementary Information. [file 41598_2023_31373_MOESM1_ESM.pdf]

Title Page

The Association between obesity and the risk of Adult Asthma: A Systematic Review and Meta-Analysis of Cohort Studies

Ganeshkumar Parasuaraman[1], Lavanya Ayyasamy[1], Dagfinn Aune[2,3,4], Abhijit Sen[5,6], Ramya Nagarajan[1], Prabhu Rajkumar[1], Saravanakumar Velusamy[1], P. Manickam[1], Satish Sivaprakasam[1]

[1] Indian Council of Medical Research- National Institute of Epidemiology, Chennai, India

[2] Department of Nutrition, Oslo New University College, Oslo, Norway

[3] Department of Epidemiology and Biostatistics, School of Public Health, Imperial College London, London, United Kingdom

[4] Department of Endocrinology, Morbid Obesity and Preventive Medicine, Oslo University Hospital, Oslo, Norway

[5] Department of Public health and Nursing, Norwegian University of Science and Technology, Trondheim, Norway

[6] Center for Oral health Services and Research (TkMidt), Trondheim, Norway

Corresponding Author:

Ganeshkumar Parasuaraman, Scientist D, Indian Council of Medical Research- National Institute of Epidemiology, Chennai, India.

Email: ganeshkumar@nieicmr.org.in| ganeshkumardr@gmail.com

Phone number: +91 9840640483

SUPPLEMENTARY APPENDIX

Supplementary Table 1: Search strategy in PubMed and EMBASE

Supplementary Table 2. List of excluded studies and exclusion reasons

Supplementary Table 3: Quality assessment

Supplementary Table 4: Relative risks from nonlinear dose-response of BMI and asthma and corresponding E-values

Supplementary Table 5: Relative Risks from nonlinear dose-response of waist circumference and asthma and corresponding E-values

Supplementary Table 6: Relative risks from nonlinear dose-response of weight gain and asthma and corresponding E-values

Supplementary Table 7: BMI and asthma, subgroup analyses

Supplementary Table 8: World Cancer Research Fund grading criteria

Supplementary Table 9: Justification for evidence grading of studies on adiposity and asthma risk

Supplementary Table 10: Evidence grading for adiposity and asthma

Supplementary Figure 1: Body mass index and asthma, high vs. low analysis

Supplementary Figure 2: Body mass index and asthma, influence analysis

Supplementary Figure 3: Funnel plot for analysis of body mass index and asthma

Supplementary Figure 4: Funnel plot for sensitivity analysis of body mass index and asthma (excluding Park et al, 2019)

Supplementary Figure 5: Waist circumference and asthma, high vs. low analysis

Supplementary Figure 6: Waist circumference and asthma, influence analysis

Supplementary Figure 7: Weight gain and asthma, high vs. low analysis

Supplementary Figure 8: Weight gain and asthma, influence analysis

Supplementary Figure 9: Weight loss and asthma, influence analysis

Supplementary Table 1: Search strategy in PubMed and EMBASE

S.NO	SEARCH TERMS in PUBMED	RESULTS
#1	**asthma[MeSH Major Topic]**<br>"asthma"[MeSH Major Topic]	108,124
#2	**((((((((((((adiposity[MeSH Terms]) OR (obesity[MeSH Terms])) OR (overweight[MeSH Terms])) OR (adipose tissue[MeSH Terms])) OR (body fat distribution[MeSH Terms])) OR (body mass index[MeSH Terms])) OR (adipose tissue hyperplasia[Title/Abstract])) OR (body weight[Title/Abstract])) OR (body composition[Title/Abstract])) OR (anthropometry[Title/Abstract])) OR (fatness[Title/Abstract])) OR (waist circumference[Title/Abstract])) OR (waist-to-hip-ratio[Title/Abstract])**<br>"adiposity"[MeSH Terms] OR "obesity"[MeSH Terms] OR "overweight"[MeSH Terms] OR "adipose tissue"[MeSH Terms] OR "body fat distribution"[MeSH Terms] OR "body mass index"[MeSH Terms] OR "adipose tissue hyperplasia"[Title/Abstract] OR "body weight"[Title/Abstract] OR "body composition"[Title/Abstract] OR "anthropometry"[Title/Abstract] OR "fatness"[Title/Abstract] OR "waist circumference"[Title/Abstract] OR "waist-to-hip-ratio"[Title/Abstract]	600,415
#3	**((((((((cohort[Title/Abstract]) OR (prospective[Title/Abstract])) OR (longitudinal[Title/Abstract])) OR (nested case-control[Title/Abstract])) OR (follow-up[Title/Abstract])) OR (relative risk[Title/Abstract])) OR (odds ratio[Title/Abstract])) OR (hazard ratio[Title/Abstract])**<br>"cohort"[Title/Abstract] OR "prospective"[Title/Abstract] OR "longitudinal"[Title/Abstract] OR "nested case control"[Title/Abstract] OR "follow-up"[Title/Abstract] OR "relative risk"[Title/Abstract] OR "odds ratio"[Title/Abstract] OR "hazard ratio"[Title/Abstract]	2,289,895
#4	**#1 AND #2 AND #3**	603
S No	**Search strategy in EMBASE**	Results
#1	'**asthma**'/exp/mj	1,77,172
#2	'**obesity**'/exp/mj OR **adiposity**:ab,ti OR **overweight**:ab,ti OR **'adipose tissue'**:ab,ti OR **'body mass'**:ab,ti OR **'body weight disorder'**:ab,ti OR **'body composition'**:ab,ti OR **anthropometry**:ab,ti OR **fatness**:ab,ti OR **fat**:ab,ti OR **'waist circumference'**:ab,ti OR **'waist-to-hip ratio'**:ab,ti OR waist-to-height ratio OR anthropometry	8,93,704
#3	**'cohort analysis'**/exp/mj OR **cohort**:ab,ti OR **'prospective study'**:ab,ti OR **'longitudinal study'**:ab,ti OR **'nested case control study'**:ab,ti OR **'relative risk'**:ab,ti OR **'odds ratio':**ab,ti **OR 'follow up'**:ab,ti OR **'hazard ratio'**:ab,ti	2,900,216
**#4**	**#1 AND #2 AND #3**	**1048**

Supplementary Table 2. List of excluded studies and exclusion reasons

Exclusion reason	Reference number
Conference abstract	1–8
Cross-sectional study design	9–21
Childhood asthma	22–26
Not relevant exposure	27
Not relevant outcome	28–40
Not relevant exposure and outcome (both are not relevant)	41–47 48–51,52
Systematic review	53,54
Unadjusted risk estimates	55,56

References:

1. Fitzpatrick S, Joks R, Shaikh W, Schneider A, Silverberg J. Obesity and super obesity are associated with increased risk of a sthma in an inner city population. *Ann Allergy, Asthma Immunol*. 2010;105(5):A38. https://www.embase.com/search/results?subaction=viewrecord&id=L70452814&from=export

2. Landen A, Goksör E, Alm B, Wennergren G. Asthma and overweight-are they connected? A prospective study from a Swedish birth cohort. *Eur Respir J*. 2017;50. doi:10.1183/1393003.congress-2017.PA1321

3. To M, Ogura N, Kono Y, et al. Obesity-associated severe asthma in east asian population. *Am J Respir Crit Care Med*. 2017;195. doi:10.1164/ajrccmconference.2017.D106

4. Rennie DC, Karunanayake C, Pahwa P, et al. Assessing associations between asthma and obesity: Measures of adiposity. *Am J Respir Crit Care Med*. 2013;187. https://www.embase.com/search/results?subaction=viewrecord&id=L71984049&from=export

5. Rael E. Obesity correlates with asthma risk. *Am J Respir Crit Care Med*. 2015;191. https://www.embase.com/search/results?subaction=viewrecord&id=L72052203&from=export

6. Song W, Kang M, Lee S, et al. Association between abdominal obesity and asthma in the elderly: Results from a community-based population study. *Allergy Eur J Allergy Clin Immunol*. 2012;67:169. doi:10.1111/all.12035

7. Khurana S, Larj MJ, Lerner LB, et al. Correlation of Body Mass Index (BMI) with asthma severity and airway inflammation. *Am J Respir Crit Care Med*. 2010;181(1).

8. Porter M, Wegienka G, Havstad S, et al. Relationship between childhood body mass index and young adult asthma. *Ann allergy, asthma Immunol Off Publ Am Coll Allergy, Asthma, Immunol*. 2012;109(6):408-411.e1. doi:10.1016/j.anai.2012.09.009

9. Aguiar FS, Rodrigues L, Ludovice M, Araujo A, Zoucas M, Cardoso AP. Obesity and asthma: Data from a difficult asthma cohort under omalizumab treatment in Rio De Janeiro, Brazil. *Am J Respir Crit Care Med*. 2014;189. https://www.embase.com/search/results?subaction=viewrecord&id=L72046553&from=export

10. Arynchyn A, Zhu S, Terry G, et al. Ct-measured visceral adiposity is associated with asthma in middle-aged women: The cardia study. *Am J Respir Crit Care Med*. 2013;187. https://www.embase.com/search/results?subaction=viewrecord&id=L71984502&from=export

11. Stanley AH, Demissie K, Rhoads GG. Asthma development with obesity exposure: Observations from the cohort of the national health and nutrition evaluation survey epidemiologic follow-up study (NHEFS). *J Asthma*. 2005;42(2):97-99. doi:10.1081/JAS-

51338

12. Sutherland ER, Lehman EB, Teodorescu M, Michael E Wechsler. Body mass index and phenotype in subjects with mild-to-moderate persistent asthma. *J Allergy Clin Immunol*. 2009;123(6):1328-34.e1. https://pubmed.ncbi.nlm.nih.gov/19501235/

13. Von Behren J, Lipsett M, Horn-Ross PL, et al. Obesity, waist size and prevalence of current asthma in the California Teachers Study cohort. *Thorax*. 2009;64(10):889-893. doi:10.1136/thx.2009.114579

14. Baltieri L, Cazzo E, de Souza AL, et al. Influence of weight loss on pulmonary function and levels of adipokines among asthmatic individuals with obesity: One-year follow-up. *Respir Med*. 2018;145:48-56. doi:10.1016/j.rmed.2018.10.017

15. Bråbäck L, Hjern A, Rasmussen F. Body mass index, asthma and allergic rhinoconjunctivitis in Swedish conscripts - A national cohort study over three decades. *Respir Med*. 2005;99(8):1010-1014. doi:10.1016/j.rmed.2005.02.004

16. Bruno A, Pace E, Cibella F, Chanez P. Body mass index and comorbidities in adult severe asthmatics. *Biomed Res Int*. 2014;2014:607192. doi:10.1155/2014/607192

17. Chandrasekhar D, Parambil AK, Puthur A, et al. An observational study to assess the prevalence and impact of obesity in asthma management. *J Young Pharm*. 2019;11(3):291-296. doi:10.5530/jyp.2019.11.58

18. Mosen DM, Schatz M, Magid DJ, Jr CAC. The relationship between obesity and asthma severity and control in adults. *J Allergy Clin Immunol*. 2008;122(3):507-11.e6. https://pubmed.ncbi.nlm.nih.gov/18774387/

19. Nystad W, Meyer HE, Nafstad P, Tverdal A, Engeland A. Body mass index in relation to adult asthma among 135,000 Norwegian men and women. *Am J Epidemiol*. 2004;160(10):969-976. doi:10.1093/aje/kwh303

20. Porter M, Wegienka G, Havstad S, et al. Relationship between childhood BMI and asthma in young adults in a birth cohort. *J Allergy Clin Immunol*. 2011;127(2):AB79. doi:10.1016/j.jaci.2010.12.323

21. Shaheen SO, Sterne JA, Montgomery SM, Azima H. Birth weight, body mass index and asthma in young adults. *Thorax*. 1999;54(5):396-402. doi:10.1136/thx.54.5.396

22. Loid P, Goksör E, Alm B, et al. A persistently high body mass index increases the risk of atopic asthma at school age. *Acta Paediatr*. 2015;104(7):707-712. doi:10.1111/apa.13015

23. Chinn S, Rona RJ. Can the increase in body mass index explain the rising trend in asthma in children? *Thorax*. 2001;56(11):845-850. doi:10.1136/thorax.56.11.845

24. Gilliland FD, Berhane K, Islam T, et al. Obesity and the risk of newly diagnosed asthma in school-age children. *Am J Epidemiol*. 2003;158(5):406-415. doi:10.1093/aje/kwg175

25. Jason E Lang, Bunnell HT, Hossain MJ, et al. Being Overweight or Obese and the Development of Asthma. *Pediatrics*. 2018;142(6). doi:10.1542/peds.2018-2119

26. Ho WC, Lin YS, Caffrey JL, et al. Higher body mass index may induce asthma among adolescents with pre-asthmatic symptoms: a prospective cohort study. *BMC Public Health*. 2011;11:542. doi:10.1186/1471-2458-11-542

27. Farina A, Crimi E, Accogli S, Camerini G, Adami GF. Preoperative assessment of respiratory function in severely obese patients undergoing biliopancreatic diversion. *Eur Surg Res Eur Chir Forschung Rech Chir Eur*. 2012;48(2):106-110. doi:10.1159/000337744

28. Acosta-Pérez E, Canino G, Ramírez R, Prelip M, Martin M, Ortega AN. Do Puerto Rican youth with asthma and obesity have higher odds for mental health disorders? *Psychosomatics*. 2012;53(2):162-171. doi:10.1016/j.psym.2011.07.011

29. Ciprandi G, Ricciardolo FLM, Signori A, et al. Increased body mass index and bronchial impairment in allergic rhinitis. *Am J Rhinol*

Allergy. 2013;27(6):e195-201. doi:10.2500/ajra.2013.27.3979

30. Sin DD, Jones RL, S F Paul Man. Obesity is a risk factor for dyspnea but not for airflow obstruction. *Arch Intern Med*. 2002;162(13):1477-1481. https://pubmed.ncbi.nlm.nih.gov/12090884/

31. Vortmann M, Eisner MD. BMI and health status among adults with asthma. *Obesity (Silver Spring)*. 2008;16(1):146-152. doi:10.1038/oby.2007.7

32. Zhang X, Hancox B, Gray A. Increasing childhood and adolescent adiposity impairs peak adult lung function. *Respirology*. 2019;24:166. doi:10.1111/resp.13492

33. Ciprandi G, Schiavetti I, Bellezza Fontana R, Sorbello V, Ricciardolo FLM. Overweight and obesity as risk factors for impaire d lung function in patients with asthma: A real-life experience. *Allergy asthma Proc*. 2014;35(4):e62-71. doi:10.2500/aap.2014.35.3773

34. Huang KHG, Lai J, Nguyen H, et al. The impact of obesity on the outcome of status asthmaticus patients admitted to the intens ive care unit. *Am J Respir Crit Care Med*. 2013;187. https://www.embase.com/search/results?subaction=viewrecord&id=L71980961&from=export

35. Lederer DJ, Wilt JS, D'Ovidio F, et al. Obesity and underweight are associated with an increased risk of death after lung transplantation. *Am J Respir Crit Care Med*. 2009;180(9):887-895. https://pubmed.ncbi.nlm.nih.gov/19608717/

36. Luthe SK, Hirayama A, Goto T, Faridi MK, Camargo CAJ, Hasegawa K. Association Between Obesity and Acute Severity Among Patients Hospitalized for Asthma Exacerbation. *J allergy Clin Immunol Pract*. 2018;6(6):1936-1941.e4. doi:10.1016/j.jaip.2018.02.001

37. Choi JH, Kim CH, Hyun IG, Kim JH, Shin TR, Park SM. Gender differentially contributes to airway hyperresponsiveness in adult asthmatics. *J Allergy Clin Immunol*. 2014;133(2):AB254. doi:10.1016/j.jaci.2013.12.904

38. S G Kapadia, Wei C, SJ B, et al. Obesity and symptoms of depression contribute independently to the poor asthma control of obesity. *Respir Med*. 2014;108(8):1100-1107. doi:10.1016/j.rmed.2014.05.012

39. Santamaria F, Montella S, Greco L, et al. Obesity duration is associated to pulmonary function impair ment in obese subjects. *Obesity (Silver Spring)*. 2011;19(8):1623-1628. doi:10.1038/OBY.2011.1

40. Sharma S, Tailor A, Warrington R, Cheang M. Is obesity associated with an increased risk for airway hyperresponsiveness and development of asthma? *Allergy, Asthma Clin Immunol*. 2008;4(2):51-58. doi:10.2310/7480.2008.00008

41. Biswas A, Kessinger CJ, Camp D, et al. Differential effects of adipose distribution and adipokine levels on airway hyper - responsiveness among HIV-infected and HIV-uninfected persons with asthma. *Am J Respir Crit Care Med*. 2015;191. https://www.embase.com/search/results?subaction=viewrecord&id=L72052578&from=export

42. Çolak Y, Afzal S, Lange P, Nordestgaard BG. High body mass index and risk of exacerbations and pneumonias in individuals with chronic obstructive pulmonary disease: observational and genetic risk estimates from the Copenhagen General Population Study. *Int J Epidemiol*. 2016;45(5):1551-1559. doi:10.1093/ije/dyw051

43. Demeyer H, Donaire-Gonzalez D, Gimeno-Santos E, et al. Physical Activity Is Associated with Attenuated Disease Progression in Chronic Obstructive Pulmonary Disease. *Med Sci Sports Exerc*. 2019;51(5):833-840. doi:10.1249/MSS.0000000000001859

44. Denton E, Bondarenko J, Tay T, et al. Factors Associated with Dysfunctional Breathing in Patients with Difficult to Treat Ast hma. *J Allergy Clin Immunol Pract*. 2019;7(5):1471-1476. doi:10.1016/j.jaip.2018.11.037

45. Dixon AE, Pratley RE, Forgione PM, et al. Effects of obesity and bariatric surgery on airway hyperresponsiveness, asthma cont rol, and inflammation. *J Allergy Clin Immunol*. 2011;128(3):502-508. doi:10.1016/j.jaci.2011.06.009

46. J B D, Chapman L, O'Brien P. Marked improvement in asthma after Lap-Band surgery for morbid obesity. *Obes Surg*. 1999;9(4):385-389. https://pubmed.ncbi.nlm.nih.gov/10484298/
47. Wan ES, Cho MH, Boutaoui N, et al. Genome-wide association analysis of body mass in chronic obstructive pulmonary disease. *Am J Respir Cell Mol Biol*. 2011;45(2):304-310. https://pubmed.ncbi.nlm.nih.gov/21037115/
48. Bahreinian S, Ball GDC, Becker AB, Kozyrskyj AL. Comorbidity with depression and overweight in children with asthma. *Allergy, Asthma Clin Immunol*. 2010;6. doi:10.1186/1710-1492-6-S2-P10
49. Contreras Z, Chen Z, Roumeliotaki T, et al. Early childhood asthma and the risk of new onset obesity: An individual participa nt meta-analysis of 16 european birth cohorts. *Am J Respir Crit Care Med*. 2018;197(MeetingAbstracts). https://www.embase.com/search/results?subaction=viewrecord&id=L622964992&from=export
50. Del Rio B, Piñeyro EE, Berber A, Sienra JL. Evaluation and comparison of metabolic syndrome in obese adolecents with and without asthma based on diet and exercise for 6 months. *Ann Allergy, Asthma Immunol*. 2009;103(5):A65. https://www.embase.com/search/results?subaction=viewrecord&id=L70254949&from=export
51. Nava FV, González AHS, Perales GM, et al. [Associations between family history of allergy, exposur e to tobacco smoke, active smoking, obesity, and asthma in adolescents]. *Arch Bronconeumol*. 2006;42(12):621-626. https://pubmed.ncbi.nlm.nih.gov/17178065/
52. Spathopoulos D, Paraskakis E, Trypsianis G, et al. The effect of obesity on pulmonary lung functi on of school aged children in Greece. *Pediatr Pulmonol*. 2009;44(3):273-280. doi:10.1002/ppul.20995
53. Camilo DF, Ribeiro JD, Toro ADC, Baracat ECE, Filho AAB. Obesity and asthma: association or coincidence? *J Pediatr (Rio J)*. 2010;86(1):6-14. https://pubmed.ncbi.nlm.nih.gov/20151086/
54. David A Beuther, Sutherland ER. Overweight, obesity, and incident asthma: a meta-analysis of prospective epidemiologic studies. *Am J Respir Crit Care Med*. 2007;175(7):661-666. doi:10.1164/rccm.200611-1717OC
55. Guerra S, Sherrill DL, Bobadilla A, Martinez FD, Barbee RA. The relation of body mass index to asthma, chronic bronchitis, and emphysema. *Chest*. 2002;122(4):1256-1263. doi:10.1378/chest.122.4.1256
56. Kronander UN, Falkenberg M, Olle Z. Prevalence and incidence of asthma related to waist circumference and BMI in a Swedish community sample. *Respir Med*. 2004;98(11):1108-1116. doi:10.1016/j.rmed.2004.03.022

Supplementary Table 3: Quality assessment

Author name	Selection of the non-exposed cohort	Ascertainment of exposure	Demonstration that outcome of interest was not present at start of study	Comparability of cohorts (adjustment for confounders)	Assessment of outcome	Was follow-up long enough for outcomes to occur	Adequacy of follow-up of cohorts	Total
Camargo et al., 1999, USA	1	1	1	2	0	1	1	7
Chen et al, 2002, Canada	1	1	1	1.5	0	1	0	5.5
Huovinen et al., 2003, Finland	1	1	1	0.75	0	1	0	4.75
Romieu et al.,2003, France	1	1	1	2	0	1	1	7
Ford et al., 2004, USA	1	1	1	1.75	0	1	1	6.75
Coogan et al, 2009, USA	1	1	1	2	0	1	0	6
Hjellvik et al., 2010, Norway	1	1	1	1.75	0.5	1	1	7.25
Korda et al.,2012, Australia	1	1	1	1.25	1	1	0	6.25
Brumpton et al., 2012, Norway	1	1	1	1.25	0	1	1	6.25
Leone et al., 2012, France	1	1	1	2	0	1	0	6
Assad et al, 2013,	1	1	1	1	0	1	0	5
Tomita et al, 2018, Japan	1	1	1	1	0.5	1	0	5.5
Park et al.,2019, Korea	1	1	1	0	0.5	1	0	4.5
Wang et al, 2020, China	1	1	1	0.75	0	1	0	4.75
Wang et al, 2021, USA	1	1	1	1.75	0	1	0	5.75

Supplementary Table 4: Relative risks from nonlinear dose-response of BMI and asthma and corresponding E-values

BMI	RR (95% CI)	E-values (lower CI)
17.5	0.98 (0.93-1.03)	1.16 (NC)
20.0	1.00	
22.5	1.09 (1.05-1.13)	1.40 (1.28)
25.0	1.23 (1.17-1.30)	1.76 (1.62)
27.5	1.43 (1.34-1.53)	2.21 (2.01)
30.0	1.69 (1.57-1.83)	2.77 (2.52)
32.5	2.03 (1.86-2.21)	3.48 (3.12)
35.0	2.45 (2.23-2.69)	4.33 (3.89)
37.5	2.97 (2.68-3.30)	5.39 (4.80)
40.0	3.64 (3.23-4.09)	6.74 (5.91)
$p_{nonlinearity}$	<0.00001	

NC: not calculable

Supplementary Table 5: Relative Risks from nonlinear dose-response of waist circumference and asthma and corresponding E-values

WC	RR (95% CI) all	E-value (lower CI)
70	1.00	1.00
75	1.08 (1.04-1.13)	1.40 (1.24)
80	1.17 (1.08-1.26)	1.67 (1.43)
85	1.25 (1.14-1.38)	1.90 (1.59)
90	1.34 (1.20-1.51)	2.15 (1.76)
95	1.44 (1.25-1.65)	2.39 (1.92)
100	1.53 (1.31-1.79)	2.64 (2.08)
105	1.63 (1.36-1.96)	2.90 (2.21)
110	1.73 (1.40-2.15)	3.19 (2.34)
$p_{nonlinearity}$	0.02	

Supplementary Table 6: Relative risks from nonlinear dose-response of weight gain and asthma and corresponding E-values

Weight gain	RR (95% CI)	E-values (lower CI)
0	1.00	
5	0.95 (0.85-1.07)	1.28 (NC)
10	1.14 (1.02-1.26)	1.54 (1.16)
15	1.36 (1.23-1.51)	2.06 (1.76)
20	1.63 (1.47-1.81)	2.64 (2.30)
25	1.96 (1.74-2.20)	3.33 (2.87)
30	2.35 (2.06-2.68)	4.13 (3.54)
$p_{nonlinearity}$	0.002	

NC: not calculable

Supplementary Table 7: BMI and asthma, subgroup analyses

	BMI and asthma, per 5 kg/m^2				
	n	RR (95% CI)	I^2 (%)	P_h[1]	P_h[2]
**All studies**	13	1.32 (1.21-1.44)	95.3	<0.0001	
**Duration of follow-up**					
<10 years follow-up	8	1.36 (1.20-1.55)	96.6	<0.0001	0.41
≥10 years follow-up	5	1.26 (1.18-1.36)	65.1	0.02	
**Sex**					
Men	6	1.15 (0.98-1.35)	77.7	<0.0001	0.20/ 0.10[3]
Women	10	1.35 (1.23-1.49)	89.0	<0.0001	
Men and women	3	1.32 (1.15-1.52)	91.1	<0.0001	
**Geographic location**					
Europe	4	1.34 (1.30-1.38)	0	0.60	0.93
North America	5	1.33 (1.17-1.50)	88.3	<0.0001	
Australia	1	1.58 (1.37-1.83)			
Asia	3	1.16 (1.03-1.32)	56.9	0.10	
**Assessment of weight and height**					
Self-reported	3	1.38 (1.19-1.60)	0	0.58	0.07
Self-reported and validated	2	1.60 (1.48-1.72)	0	0.87	
Measured	8	1.25 (1.14-1.37)	95.9	<0.0001	
**Number of cases**					
Cases <250	3	1.32 (1.13-1.54)	0	0.86	0.92
Cases 250-999	4	1.32 (1.13-1.53)	98.6	<0.0001	
Cases ≥1000	6	1.32 (1.19-1.46)	76.1	0.001	
**Study quality**					
0-3 stars	0				0.02
>3-6 stars	7	1.22 (1.11-1.33)	88.6	<0.0001	

>6-8 stars		6	1.42 (1.31-1.54)	73.7	0.002	
**Adjustment for confounding factors**						
Age	Yes	13	1.32 (1.21-1.44)	95.3	<0.0001	NC
	No	0				
Family history of asthma	Yes	2	1.44 (1.22-1.69)	73.8	0.05	0.29
	No	11	1.30 (1.18-1.43)	95.5	<0.0001	
Allergy	Yes	3	1.28 (1.13-1.44)	0	0.80	0.79
	No	10	1.33 (1.21-1.47)	96.4	<0.0001	
Education	Yes	4	1.36 (1.28-1.44)	43.2	0.15	0.43
	No	9	1.30 (1.16-1.44)	93.5	<0.0001	
Income	Yes	4	1.31 (1.12-1.53)	95.7	<0.0001	0.77
	No	9	1.33 (1.23-1.43)	78.3	<0.0001	
Alcohol	Yes	4	1.33 (1.04-1.69)	89.8	<0.0001	0.83
	No	9	1.32 (1.24-1.41)	78.4	<0.0001	
Smoking	Yes	12	1.33 (1.21-1.45)	95.7	<0.0001	0.79
	No	1	1.26 (1.00-1.60)			
Physical activity	Yes	8	1.30 (1.17-1.44)	97.0	<0.0001	0.54
	No	5	1.48 (1.23-1.53)	26.9	0.24	

Abbreviations: NC not calculable because no studies were present in one of the subgroups; RRs relative risk estimates.

n denotes the number of studies included in each subgroup analysis

[1] P-value for heterogeneity within each subgroup

[2] P-value for heterogeneity between subgroups with meta-regression analysis

[3] P-value for heterogeneity between men and women with meta-regression analysis (excluding studies of men and women combined)

Supplemental Table 8. World Cancer Research Fund grading criteria

Grading	Criteria
Convincing	A convincing relationship should be robust enough to be highly unlikely to be modified in the foreseeable future as new evidence accumulates. All of the following are generally required:<br>- Evidence from more than one study type<br>- Evidence from at least two independent cohort studies<br>- No substantial unexplained heterogeneity within or between study types or in different populations relating to the presence or absence of an association, or direction of effect<br>- Good quality studies to exclude with confidence the possibility that the observed association results from random or systematic error, including confounding, measurement error, and selection bias<br>- Presence of a plausible biological gradient in the association. Such a gradient need not be linear or even in the same direction across different levels of exposure, so long as this can be explained plausibly<br>- Strong and plausible experimental evidence, either from human studies or relevant animal models, that typical human exposures can lead to relevant outcomes
Probable	All of the following are generally required:<br>- Evidence from at least two independent cohort studies, or at least five case-control studies<br>- No substantial unexplained heterogeneity within or between study types or in different populations relating to the presence or absence of an association, or direction of effect<br>- Good quality studies to exclude with confidence the possibility that the observed association results from random or systematic error, including confounding, measurement error, and selection bias<br>- Evidence for biological plausibility
Limited - suggestive	All of the following are generally required:<br>- Evidence from at least two independent cohort studies, or at least five case-control studies<br>- The direction of effect is generally consistent though some unexplained heterogeneity may be present<br>- Evidence for biological plausibility
Limited - no conclusion	Evidence is so limited that no firm conclusion can be made, but this does not mean that there is evidence of no relationship. The evidence might be graded "limited - no conclusion" for several reasons:<br>- limited number of studies<br>- inconsistency of direction of effect<br>- poor quality of studies (e.g. lack of adjustment for known confounders)<br>- or any combination of these factors
Substantial effect on risk unlikely	All of the following are generally required:<br>- Evidence from more than one study type<br>- Evidence from at least two independent cohort studies

	- Summary estimate of effect close to 1.0 for comparison of high versus low exposure categories<br>- No substantial unexplained heterogeneity within or between study types or in different populations<br>- Good quality studies to exclude with confidence the possibility that the absence of association results from random or systematic error, including inadequate power, imprecision or error in exposure measurement, inadequate range of exposure, confounding, and selection bias<br>- Absence of a demonstrable biological gradient (dose response)<br>- Absence of strong and plausible experimental evidence, either from human studies or relevant animal models, that typical human exposures lead to relevant outcomes

Specific upgrading factors:

1) Presence of a plausible biological gradient (dose response) in the association. Such a gradient need not be linear or even in the same direction across the different levels of exposure, so long as this can be explained plausibly.

2) A particularly large summary effect size (an odds ratio or relative risk of 2.0 or more, depending on the unit of exposure) after appropriate control for confounders.

3) Evidence from randomised trials in humans.

4) Evidence from appropriately controlled experiments demonstrating one or more plausible and specific mechanisms actually operating in humans.

5) Robust and reproducible evidence from experimental studies in appropriate animal models showing that typical human exposures can lead to relevant health outcomes.

Supplemental Table 9. Justification for evidence grading of studies on adiposity and asthma risk

Requirements for grading of convincing	BMI	Waist circumference	Weight gain	Weight loss
Statistically significant and robust association	Statistically significant strong positive association in high vs. low, linear and nonlinear dose-response analyses. Association is robust in influence analyses.	Statistically significant strong positive association in high vs. low, linear and nonlinear dose-response analyses. The association is robust in influence analyses.	Statistically significant strong positive association in high vs. low, linear and nonlinear dose-response analyses. The association is robust in influence analyses.	Non-significant weak inverse association, which is not substantially altered in influence analyses.
Evidence from at least two independent cohort studies	13 cohort studies	4 cohort studies	4 cohort studies	4 cohort studies
No substantial unexplained heterogeneity within or between study types or in different populations relating to the presence or absence of an association, or direction of effect	There is high heterogeneity overall, but this is with regard to the strength of the association more than the direction of the association. All studies reported risk estimates in the direction of increased risk. Lower heterogeneity in some subgroups. Consistent findings across geographic regions.	There is high heterogeneity overall, but this is with regard to the strength of the association more than the direction of the association. All studies reported risk estimates in the direction of increased risk.	There is high heterogeneity overall, but this is with regard to the strength of the association more than the direction of the association. All studies reported risk estimates in the direction of increased risk.	There is high heterogeneity overall, but this is with regard to the strength of the association more than the direction of the association. All studies reported risk estimates in the direction of increased risk.
Good quality studies to exclude with confidence the possibility that the observed association results from random or systematic error, including confounding,	Moderately high study quality.<br><br>Publication bias is explained by one large study with a lower estimate than the remaining studies which is driving the asymmetry in the funnel plot. Little indication of	Moderately high study quality.<br><br>Too few studies to test for publication bias and for meaningful subgroup analyses.<br><br>All studies excluded subjects with prevalent	Moderately high study quality.<br><br>Too few studies to test for publication bias and for meaningful subgroup analyses.<br><br>All studies excluded subjects with prevalent	Moderately high study quality.<br><br>Too few studies to test for publication bias and for meaningful subgroup analyses.<br><br>All studies excluded subjects with prevalent

measurement error, and selection bias	between subgroup heterogeneity. Stronger association in studies with high quality.<br><br>Somewhat stronger association in studies with self-reported weight and height compared to studies with measured weight and height (although no significant between subgroup heterogeneity), however, the association is also significant among studies with measured data.<br><br>All studies excluded subjects with prevalent asthma at baseline. Exposed and non-exposed participants were selected from the same populations.	asthma at baseline. Exposed and non-exposed participants were selected from the same population.	asthma at baseline. Exposed and non-exposed participants were selected from the same population.	asthma at baseline. Exposed and non-exposed participants were selected from the same population.
Presence of a plausible biological gradient in the association. Such a gradient need not be linear or even in the same direction across different levels of exposure, so long as this can be explained plausibly	Evidence of a strong dose-response relationship, with increased risk above a BMI of 18-20 ($p_{nonlinearity}$<0.0001).	Evidence of a strong dose-response relationship, with increased risk above 70 cm ($p_{nonlinearity}$=0.02).	Evidence of a strong dose-response relationship, with increased risk from 10 kg of weight gain ($p_{nonlinearity}$=0.002).	Dose-response analyses were not possible because there was only one category of weight loss across studies.

Strong and plausible experimental evidence, either from human studies or relevant animal models, that typical human exposures can lead to relevant outcomes	There is evidence for plausible mechanisms including studies showing narrowing airways, reduced lung volume, and reduced lung function with higher BMI. There is some indication from cohorts and trials that weight loss among obese subjects can improve lung function.	There is evidence for plausible mechanisms including studies showing narrowing airways, reduced lung volume, and reduced lung function with higher BMI. There is some indication from cohorts and trials that weight loss among obese subjects can improve lung function.	There is evidence for plausible mechanisms including studies showing narrowing airways, reduced lung volume, and reduced lung function with higher BMI and these are likely to extend to weight gain. There is some indication from cohorts and trials that weight loss among obese subjects can improve lung function.	There is evidence for plausible mechanisms including studies showing narrowing airways, reduced lung volume, and reduced lung function with higher BMI and these are likely to extend to weight gain. There is some indication from cohorts and trials that weight loss among obese subjects can improve lung function.
Final grading and justification for overall assessment.	Convincing evidence that higher BMI increases the risk of asthma.<br><br>Justification: Strong positive associations observed across a large number of cohort studies, which are significant across high vs. low, linear and nonlinear dose-response analyses. Results are consistent results across regions and robust in influence analyses. Although heterogeneity is high, all studies report effect estimates in the direction of increased risk. Although Egger's test indicates possible publication bias, the test	Limited-suggestive evidence that higher waist circumference increases the risk of asthma.<br><br>Justification: Strong positive associations observed across four cohort studies which are significant across all analyses. Too few studies to conduct meaningful subgroup analyses and to test for publication bias. The results are robust in influence analyses. Although heterogeneity is high, all studies report effect estimates in the direction of increased	Limited-suggestive evidence that higher weight gain increases the risk of asthma.<br><br>Justification: Strong positive associations observed across four cohort studies which are significant across all analyses. Too few studies to conduct meaningful subgroup analyses and to test for publication bias. The results are robust in influence analyses. Although heterogeneity is high, all studies report effect estimates in the direction of increased risk. Biologically plausible mechanisms exist for	Limited – no conclusion evidence for an association between weight loss and asthma.<br><br>Justification: Non-significant inverse association based on four cohort studies. Too few studies to conduct meaningful subgroup analyses and to test for publication bias. The results do not change in influence analyses. There is no heterogeneity. Biologically plausible mechanisms exist for general obesity are likely to apply also for weight gain.

	and the asymmetry in the funnel plot is explained by one large study which shows a much weaker association than the remaining studies. Biologically plausible mechanisms exist.	risk. Biologically plausible mechanisms exist for general obesity and may also apply to waist circumference.	general obesity are likely to apply also for weight gain.	

Supplemental Table 10. Evidence grading for adiposity and asthma

	Reduced risk	Increased risk
Convincing	-	BMI
Probable	-	-
Limited-suggestive	-	Waist circumference, weight gain
Limited - no conclusion	Weight loss	

Supplementary figure 1. Body mass index and asthma, high vs. low analysis

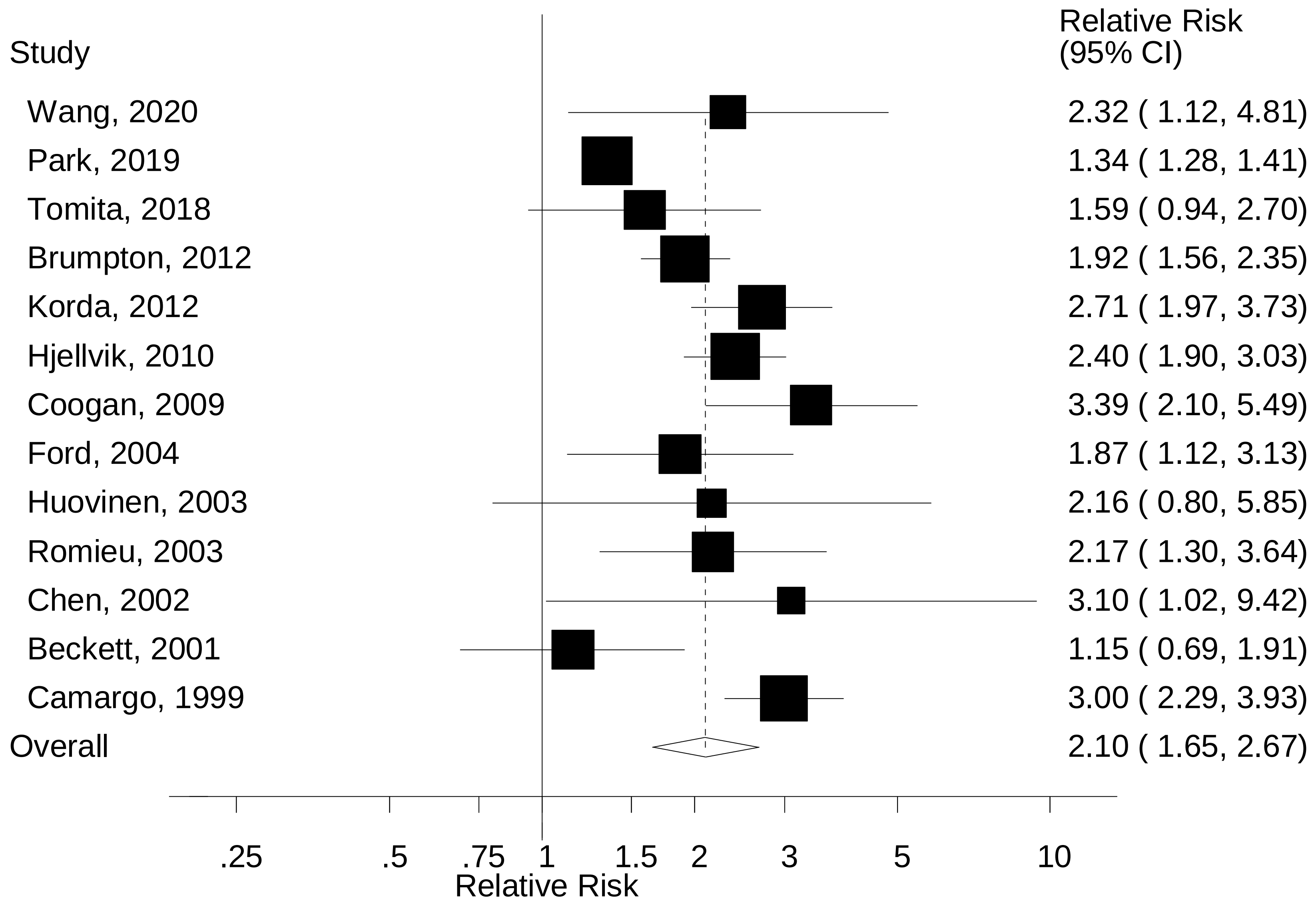

Supplementary Figure 2. Body mass index and asthma, influence analysis

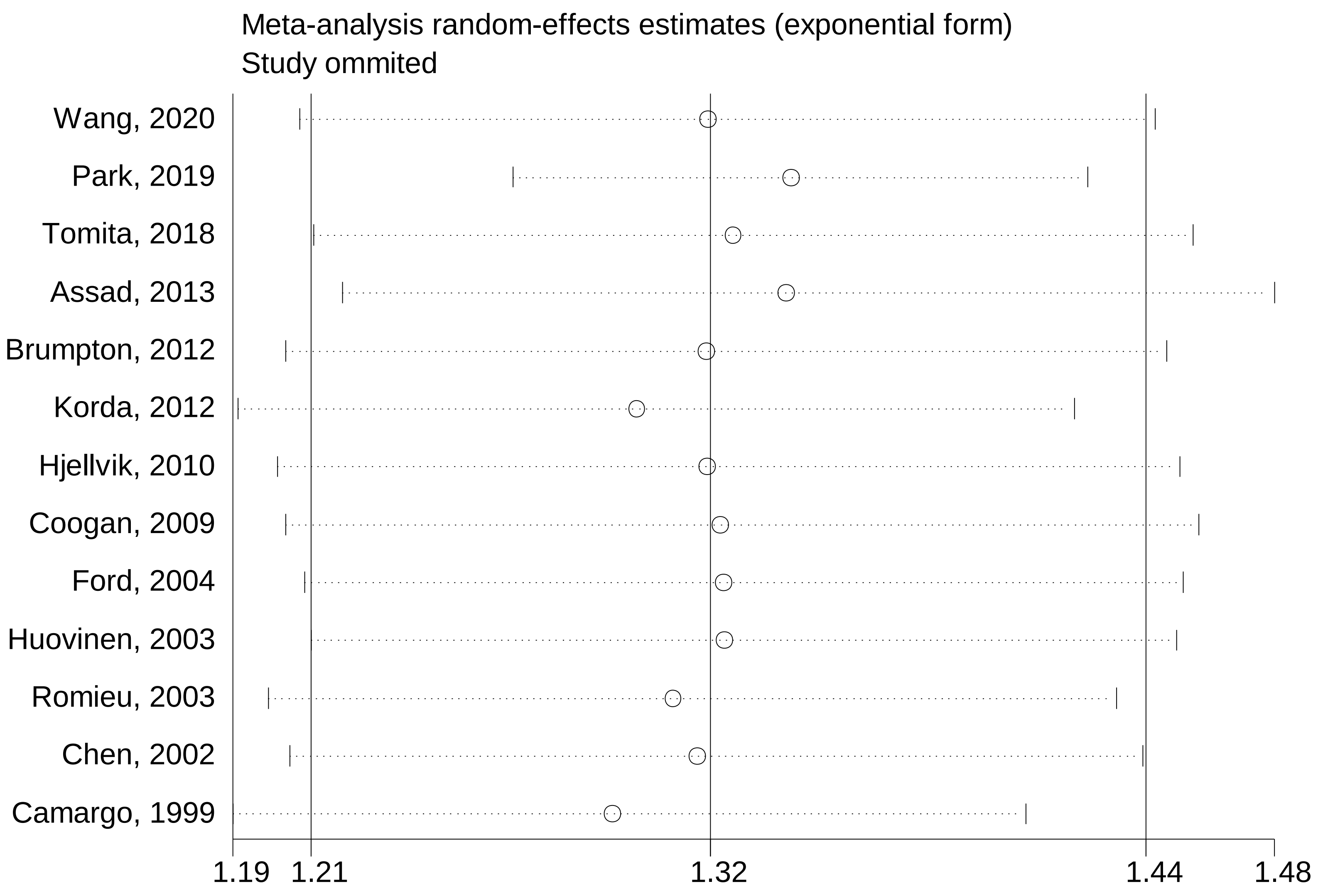

**Study omitted**	**RR (95% CI)**
Wang, 2020	1.32 (1.20-1.44)
Park, 2019	1.34 (1.26-1.42)
Tomita, 2018	1.32 (1.21-1.45)
Assad, 2013	1.34 (1.22-1.47)
Brumpton, 2012	1.32 (1.20-1.44)
Korda, 2012	1.30 (1.19-1.42)
Hjellvik, 2010	1.32 (1.20-1.45)
Coogan, 2009	1.32 (1.20-1.45)
Ford, 2004	1.32 (1.20-1.45)
Huovinen, 2003	1.32 (1.21-1.45)
Romieu, 2003	1.31 (1.19-1.43)
Chen, 2002	1.31 (1.20-1.44)
Camargo, 1999	1.29 (1.18-1.40)
**Combined**	**1.32 (1.21-1.44)**

Supplementary Figure 3. Funnel plot for analysis of body mass index and asthma

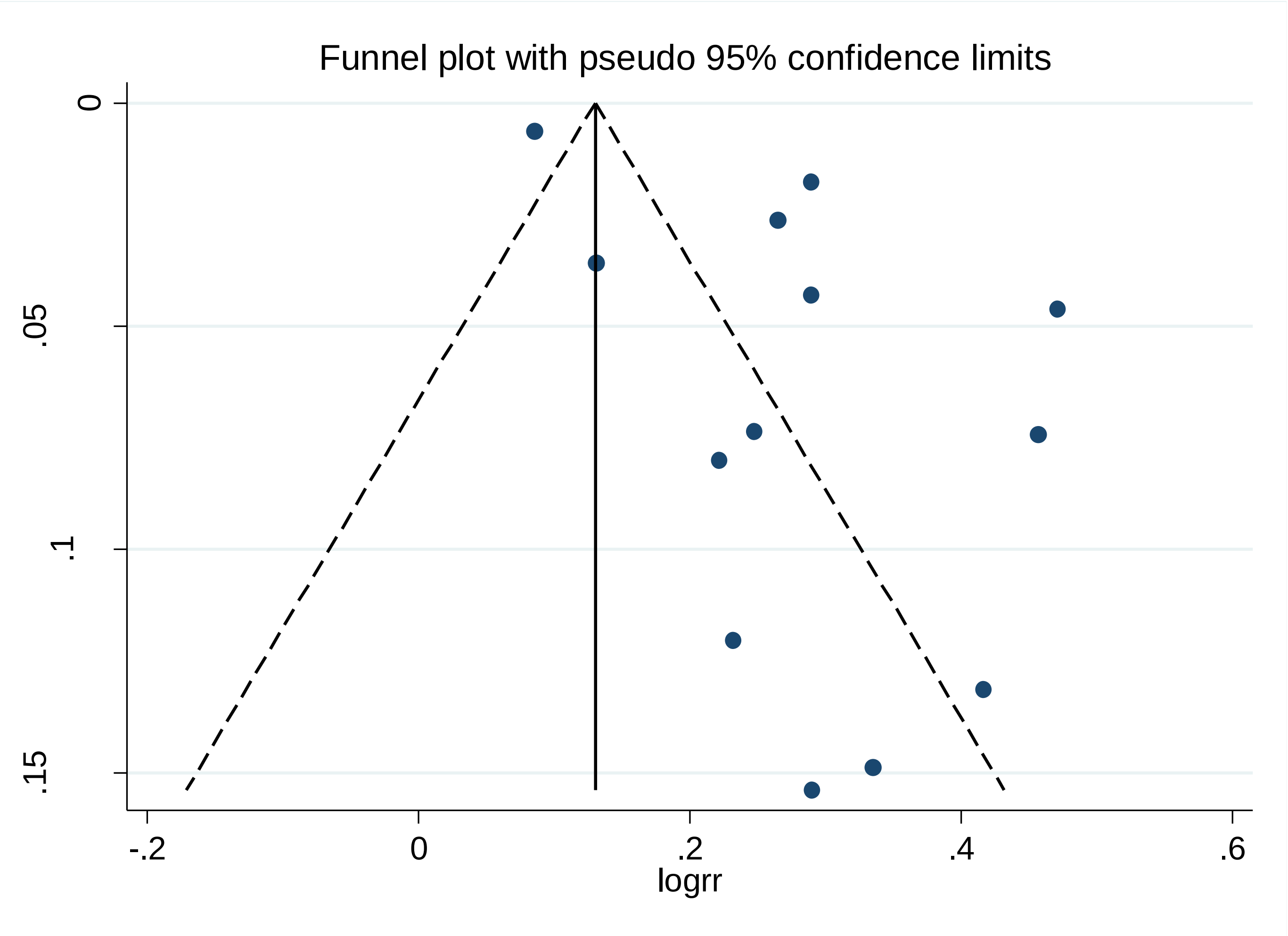

Supplementary Figure 4. Funnel plot for sensitivity analysis of body mass index and asthma (excluding Park et al, 2019)

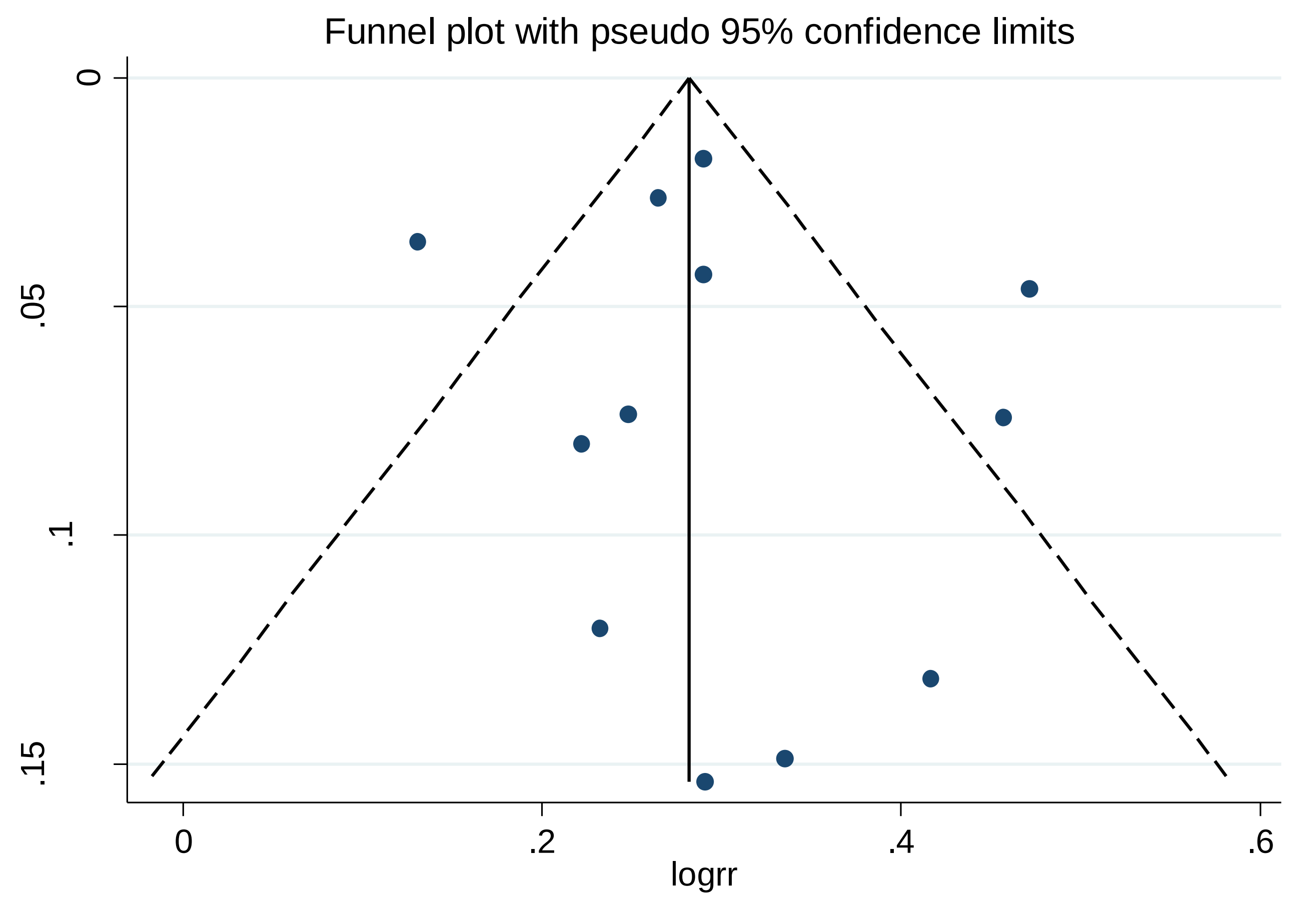

Supplementary Figure 5. Waist circumference and asthma, high vs. low analysis

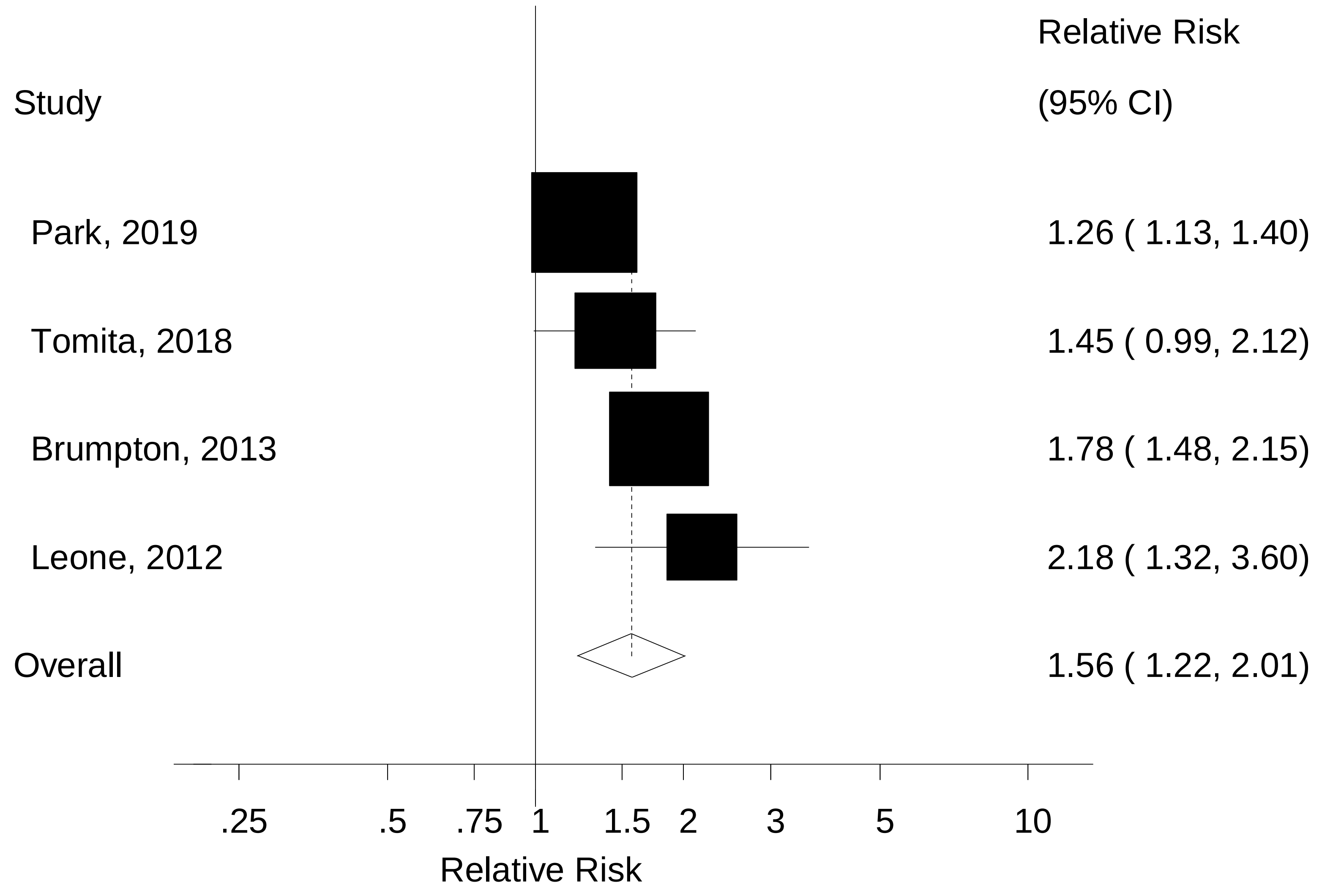

Supplementary Figure 6. Waist circumference and asthma, influence analysis

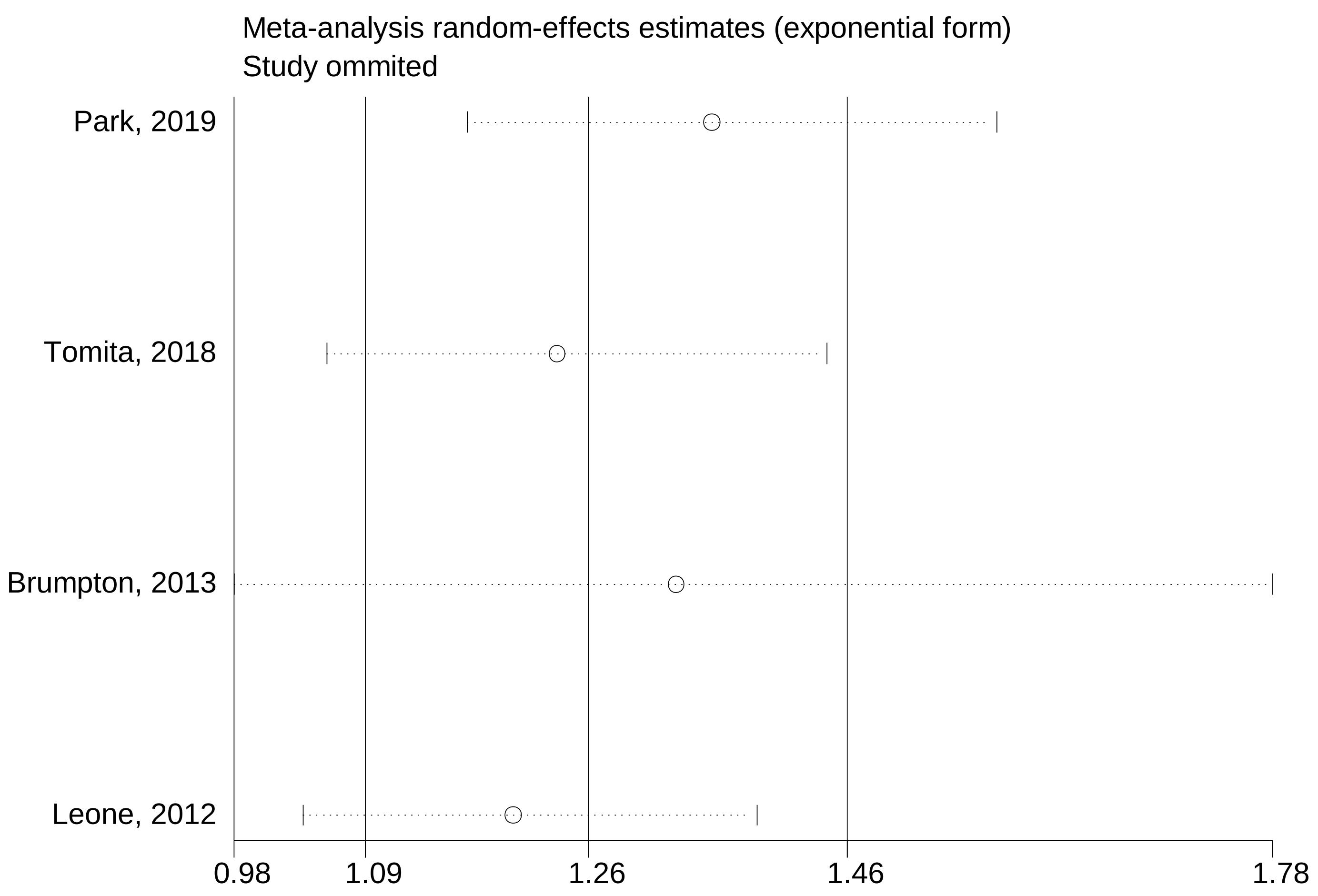

Study omitted	RR (95% CI)
Park, 2019	1.35 (1.16-1.57)
Tomita, 2018	1.23 (1.05-1.44)
Brumpton, 2013	1.32 (0.98-1.78)
Leone, 2012	1.19 (1.03-1.38)
**Combined**	**1.25 (1.08-1.45)**

Supplementary Figure 7. Weight gain and asthma, high vs. low analysis

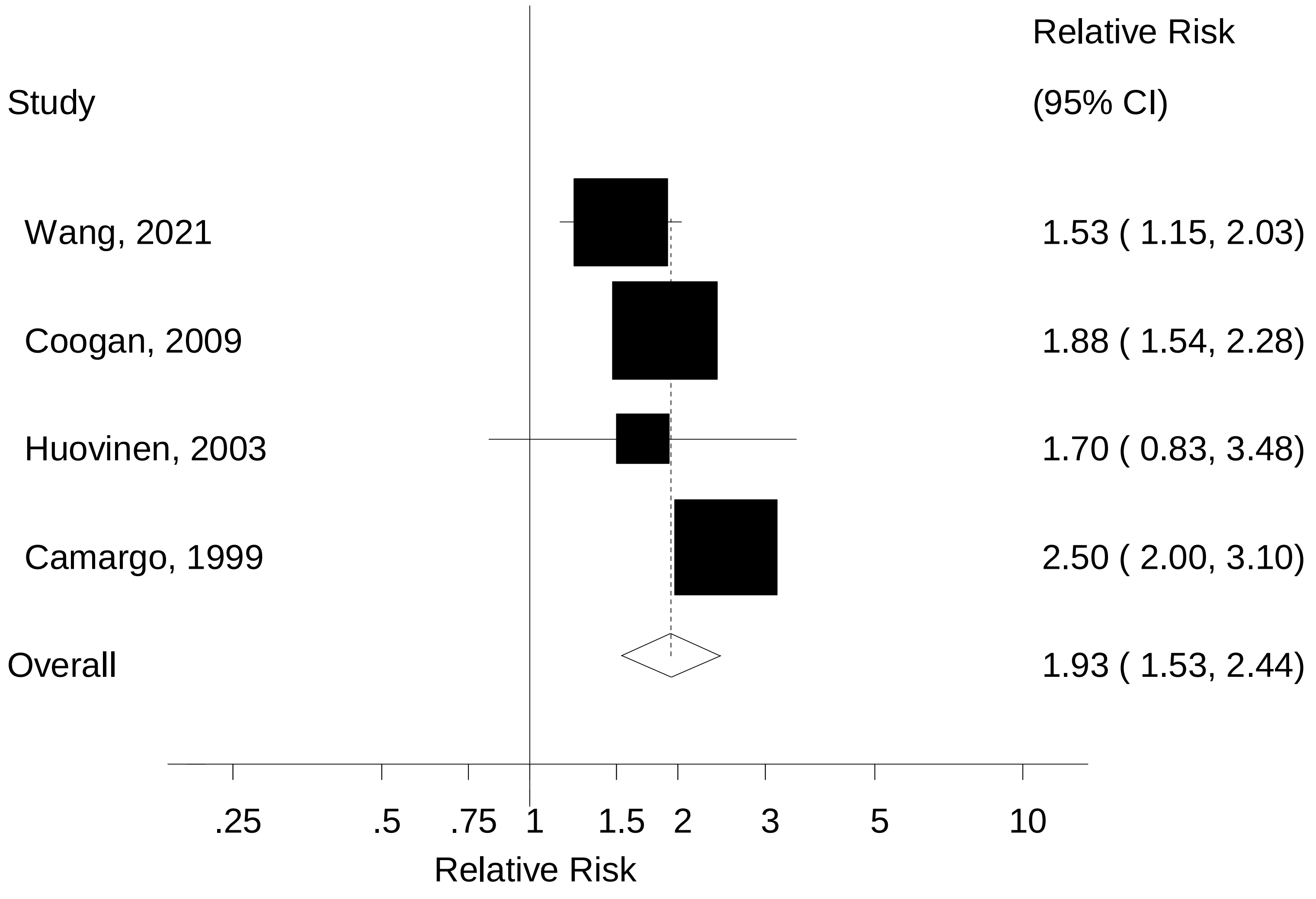

Supplementary Figure 8. Weight gain and asthma, influence analysis

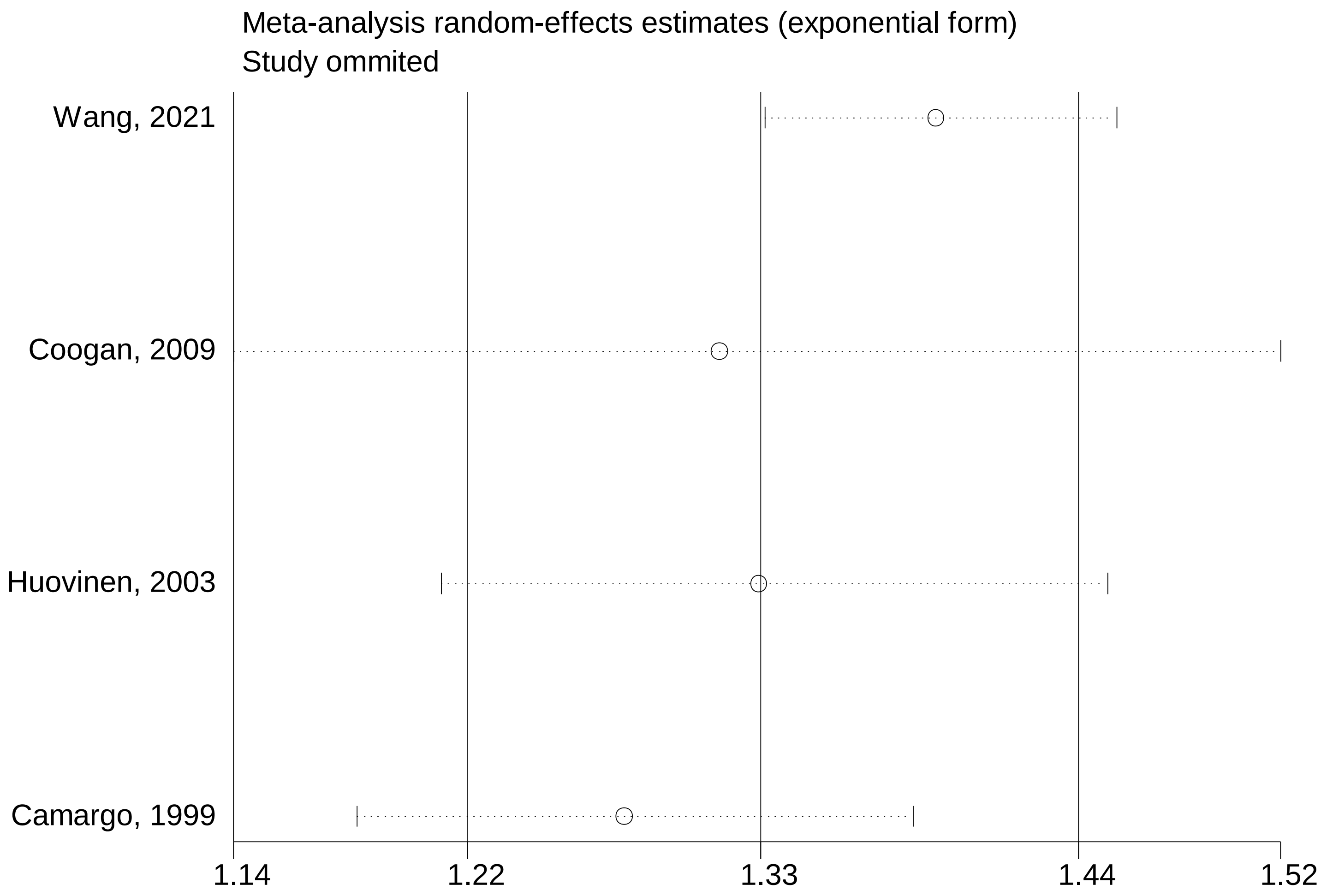

**Study omitted**	**RR (95% CI)**
Wang, 2021	1.39 (1.32-1.45)
Coogan, 2009	1.31 (1.13-1.51)
Huovinen, 2003	1.32 (1.21-1.45)
Camargo, 1999	1.27 (1.18-1.38)
**Combined**	**1.32 (1.22-1.44)**

Supplementary Figure 9. Weight loss and asthma, influence analysis

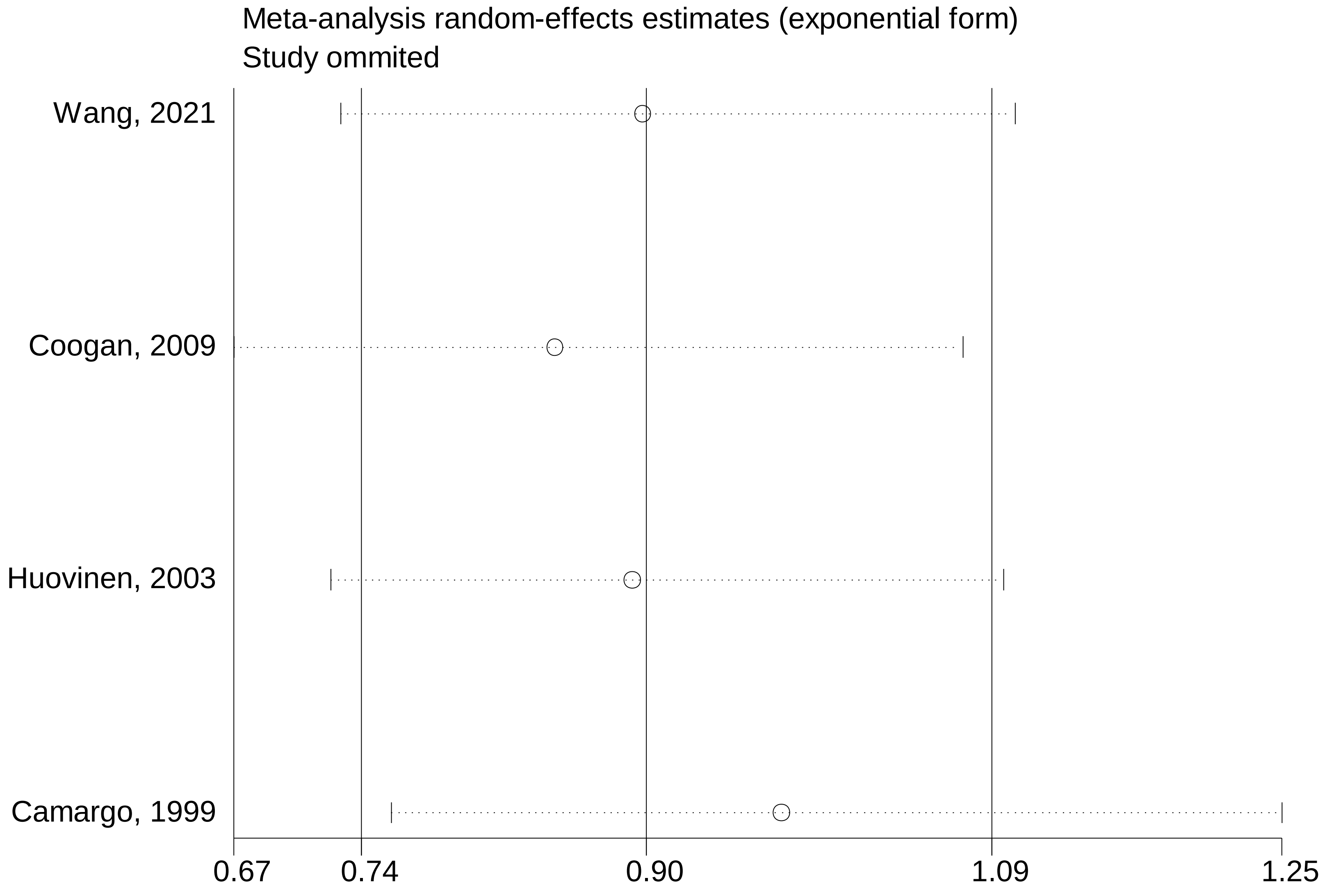

\

Study omitted	RR (95% CI)
Wang, 2021	0.89 (0.72-1.10)
Coogan, 2009	0.84 (0.67-1.07)
Huovinen, 2003	0.89 (0.72-1.09)
Camargo, 1999	0.97 (0.75-1.25)
**Combined**	**0.89 (0.74-1.09)**
